# Common weeds as heavy metal bioindicators: a new approach in biomonitoring

**DOI:** 10.1038/s41598-023-34019-9

**Published:** 2023-04-28

**Authors:** A. Cakaj, M. Lisiak-Zielińska, A. Hanć, A. Małecka, K. Borowiak, M. Drapikowska

**Affiliations:** 1 Department of Ecology and Environmental Protection, Faculty of Environmental and Mechanical Engineering, Poznań University of Life Sciences, Piątkowska 94C, 60-649 Poznań, Poland; 2 Department of Trace Analysis, Faculty of Chemistry, Adam Mickiewicz University, Uniwersytetu Poznańskiego 8, 61-614 Poznań, Poland; 3 The Cancer Epidemiology and Prevention Unit, Greater Poland Cancer Centre, Garbary 15 Street, 61-866 Poznan, Poland

**Keywords:** Environmental sciences, Ecology

## Abstract

Environmental pollution by heavy metals affects both urban and non-urban areas of Europe and the world. The use of bioindicator plants for the detection of these pollutants is a common practice. An important property of potential bioindicators is their easy availability and wide distribution range, which means that they can be practically used over a wide area. Therefore, common and widely distributed weeds: *Trifolium pratense* L., *Rumex acetosa* L., *Amaranthus retroflexus* L., *Plantago lanceolata* L., ornamental species *Alcea rosea* L., and *Lolium multiflorum* L. var. Ponto were selected as a potential bioindicators of heavy metals (Cd, Pb, Cu, Zn). Plants were exposed in the same soil conditions in three sample sites in the Poznań city. It was found that all species had heavy metal accumulation potential, especially *A. rosea*, *P. lanceolata* and *L. multiflorum* for Zn (BCF = 6.62; 5.17; 4.70) and *A. rosea, P. lanceolata* for Cd (BCF = 8.51; 6.94). Translocation of Cu and Zn was the most effective in *T. pratense* (TF_Cu_ = 2.55; TF_Zn_ = 2.67) and in *A. retroflexus* (TF_Cu_ = 1.50; TF_Zn_ = 2.23). Cd translocation was the most efficient in *T. pratense* (TF_Cd_ = 1.97), but PB was the most effective translocated in *A. retroflexus* (TF_Pb_ = 3.09).. Based on physiological response to stress, it was detected an increasing level of hydrogen peroxide (H_2_O_2_) in roots and leaves of all samples, with the highest in all organs of *A. rosea*. Enzymatic activity levels of CAT, APOX, and also the marker of polyunsaturated fatty acid peroxidation MDA, were higher after 6 weeks of exposure in comparison to control samples and varied in time of exposure and between species and exposure. After the experiment, in almost all samples we detected a reduction of chlorophyll content and relative water content, but in efficiency of photosynthesis parameters: net photosynthesis rate, intercellular CO_2_ concentration and stomatal conductance, we noted increased values, which proved the relatively good condition of the plants. The examined weeds are good bioindicators of heavy metal contamination, and their combined use makes it possible to comprehensively detection of environmental threats.

## Introduction

With the intensive development of human activities, urban areas have rapidly undergone significant and rapid changes. In urban areas, one of the most important urban pollutants are metals and metalloids^1, 2^. Metals and metalloids are the subject of numerous studies because they are persistent and among the most widely disseminated industrial pollutants^3^. The main sources of these elements are natural sources, such as natural weathering of crust, erosion, and anthropogenic activities, like urban runoff, agricultural and industrial activities, and many others^4^. Exposure to heavy metals usually has subtle and chronic symptoms, moreover, exposure to airborne metals induces physiological responses in organisms and broad health effects in humans^5^. Also, the contamination of dietary substances by heavy metals is known to have a range of adverse effects on humans, animals and plants^6, 7^. In plants, their toxicity varies depending on the specific metal, plant spieces, pH, soil composition, and chemical form. Certain heavy metals are considered to be essential for development and plant growth^8^. However, excess amounts of these elements can become toxic to plants^9^, thus affecting plants only negatively^10^.

The exposure of plants to unfavorable environmental conditions, including at higher concentrations of heavy metals, can cause an increase in the production of reactive oxygen species (ROS) such as singlet oxygen [(1) O_2_], superoxide [(O_2_)^−.^)], hydrogen peroxide (H_2_O_2_), and hydroxyl radical (OH^.^). ROS modifies proteins, damages DNA and causes free radical oxidation of unsaturated fatty acids or other lipids the product of which is MDA. The ROS detoxification process in plants is essential for the protection of plant cells, and therefore it seems that metal hyperaccumulating plants should have extremely efficient antioxidative and detoxicative defense mechanisms, enabling growth and development in a polluted environment^11^. Plant responses and tolerance to heavy metal stress are dependent on enzymatic antioxidants comprising ascorbate peroxidase (APOX), catalase (CAT), and the final product of polyunsaturated fatty acid peroxidation—malondialdehyde (MDA). These proteins take part in ROS detoxification in plants^12^, and are present in practically all subcellular compartments. Usually, an organelle has more than one enzyme able to scavenge a single ROS^13^. As a result of oxidative stress, photosynthetic processes are disturbed, from electron transport to carbon bonding. Limitation of any of these processes within the photosynthetic apparatus reduces the ability of the chloroplast membrane to absorb light energy, increasing the ability to form oxidative radicals in the chloroplast, and as a consequence limits the productivity of photosynthesis^14^.

Identifying areas with higher concentrations of heavy metals, guidelines, and effective legislation are necessary. In addition, these metals should be subject to mandatory monitoring due to their toxicity and possible bioaccumulation^4^. To control pollutants is a complex issue: the origin of pollutants and emission must be identified, critical emissions must be controlled, techniques must be developed that are sufficiently sensitive and low-cost to allow simultaneous measurement of multiple contaminants, risks and economic factors must be considered^15^. One inexpensive and simple method to determine the heavy metal concentration in the air and obtain information associated with the population’s exposure to air pollutants in a particular ecosystem is biomonitoring^16^. Moreover, to obtain information about the changes in ecosystems, bioindicators can be used. Some plants are well known for their ability to accumulate trace elements from the environment. Therefore, they have been used in a number of monitoring investigations, providing low-cost information regarding environmental quality with the advantage of easy sampling. Various studies have used as bioindicators herbaceous plants (e.g., *Taraxacum officinale*, L., *Carduus nutans* L., *Plantago major* L., *Urtica dioica* L.), which are more common in urban environments (e.g.^17–20^).

Previous studies have indicate the ability of selected species to bioaccumulate heavy metals. However, so far, they have not been studied simultaneously, under the same contamination conditions, as well as taking into account the physiological response. The control of toxic elements contamination with the simultaneous use of commonly available weeds like *Plantago lanceolata* L.^21^, *Amaranthus retroflexus* L.^22^, *Trifolium pratense* L.^1^, *Rumex acetosa*^23^ and also known for the ability of trace metals accumulation, old ornamental plant (*Alcea rosea*)^24^, seems to be a necessary procedure that enables comprehensive estimation of trace metals environment contamination. Selected weeds have the basic characteristics of bioindicators, such as: long life cycle, wide geographic ranges, large numbers of occurrence and ease of determination. Our research focused on checking whether selected weed species react in a characteristic way to changes in the environment (reaction to physical and chemical stress) depending on the place of occurrence. We made these arrangements by using the active bioindication based on the exposure and observation of specific plant species. This is where the purpose of our research lies—to evaluate widespread and very common bioindicators.

Considering the above, the main objectives of this study were as follows: (i) to determine the accumulation level of trace metals (Cu, Zn, Cd and Pb) in selected plant species exposed in even soil conditions in three research sites in the city; (ii) to assess the bioaccumulation potential of examined species; (iii) to establish translocation of metals from soil to above-ground parts; (iv) to study the physiological conditions of plants; (v) to determine the activity of oxidative stress parameters; and (vi) to assess the concentrations of enzymes of the antioxidative system.

## Results

The content of copper, zinc, cadmium and lead in the soil used for pot culture and in the tissues (roots and leaves) of all samples of the studied species was examined, and then bioconcentration and translocation factors were calculated based on these results to assess bioaccumulation potential of examined species. Moreover, the plant physiological responses for stress were detected for all samples of examined species.

### Heavy metal contents

The heavy metal contents for all species at all research sites showed the following tendency: Zn > Cu > Pb > Cd. This tendency was found for soil and plant organs (roots and leaves). In addition, for zinc and cadmium, the lowest values were mostly observed in the soil, while for copper and lead their content was generally the highest in the soil, with only a few exceptions (Suppl. Table S1). Analyzing the data in more detail, it was found that Cu, Zn, Cd and Pb concentrations in roots and leaves differ in all species. The highest Cu concentration in roots was found in *T. pratense* (2C: 20.38 mg kg^−1^); also a high value was recorded in *R. acetosa* (3C: 10.51 mg kg^−1^) and in *L. multiflorum* (1B: 8.30 mg kg^−1^). The highest Cu accumulation in leaves was detected in *R. acetosa* (3C: 9.66 mg kg^−1^), in *T. pratense* (2B: 9.20 mg kg^−1^) and in *A. rosea* (4B: 8.13 mg kg^−1^). The highest Zn concentration in roots was detected in *L. multiflorum* (1C: 81.13 mg kg^−1^), *P. lanceolata* (6C: 80.45 mg kg^−1^), *T. pratense* (2C: 68.49 mg kg^−1^), *A. rosea* (4B: 55.73 mg kg^−1^) and *A. retroflexus* (5A: 52.62 mg kg^−1^). In *L. multiflorum* leaves the highest Zn concentration (1B: 172.45 mg kg^−1^) was noted; high Zn concentration in leaves of *A. rosea* (4A: 135.85 mg kg^−1^) and *P. lanceolata* (6C: 114.77 mg kg^−1^) was also found. In the soil samples Zn concentration was lower than in plant tissues. The Cd amount varied in roots and leaves of studied species. In roots of *P. lanceolata* (Control: 0.69 mg kg^−1^) we found the highest Cd concentration; also high Cd concentration was found in *A. rosea* (4A and 4B: 0.58 mg kg^−1^) roots. In leaves of *A. rosea* (4B: 1.24 mg kg^−1^) we observed the highest Cd amount; also in *L. multiflorum* leaves (1C: 0.79 mg kg^−1^) and in *P. lanceolata* (6A: 1.11 mg kg^−1^) high Cd concentration was detected in leaves. The highest Pb amount was found in *L. multiflorum* roots (1C: 1.32 mg kg^−1^) as well as high Pb concentration in roots of *R. acetosa* (3C: 0.75 mg kg^−1^). In leaf tissue of *L. multiflorum* the highest Pb concentration was detected (1A: 1.21 mg kg^−1^), in *R. acetosa* Pb concentration in leaves reached 0.99 mg kg^−1^, and in *P. lanceolata* it reached 0.77 mg kg^−1^. Pb concentration in soil samples was higher than in plants. However, two-way ANOVA of species and site effect revealed significant influence (α ≤ 0.05) of both factors on all analyzed trace elements levels in roots and leaves. The both factors were found to have no significant effect on the analyzed levels of these elements in soil, except of cadmium (one outlier observation in control) (Suppl. Table S2).

**Figure 1:**
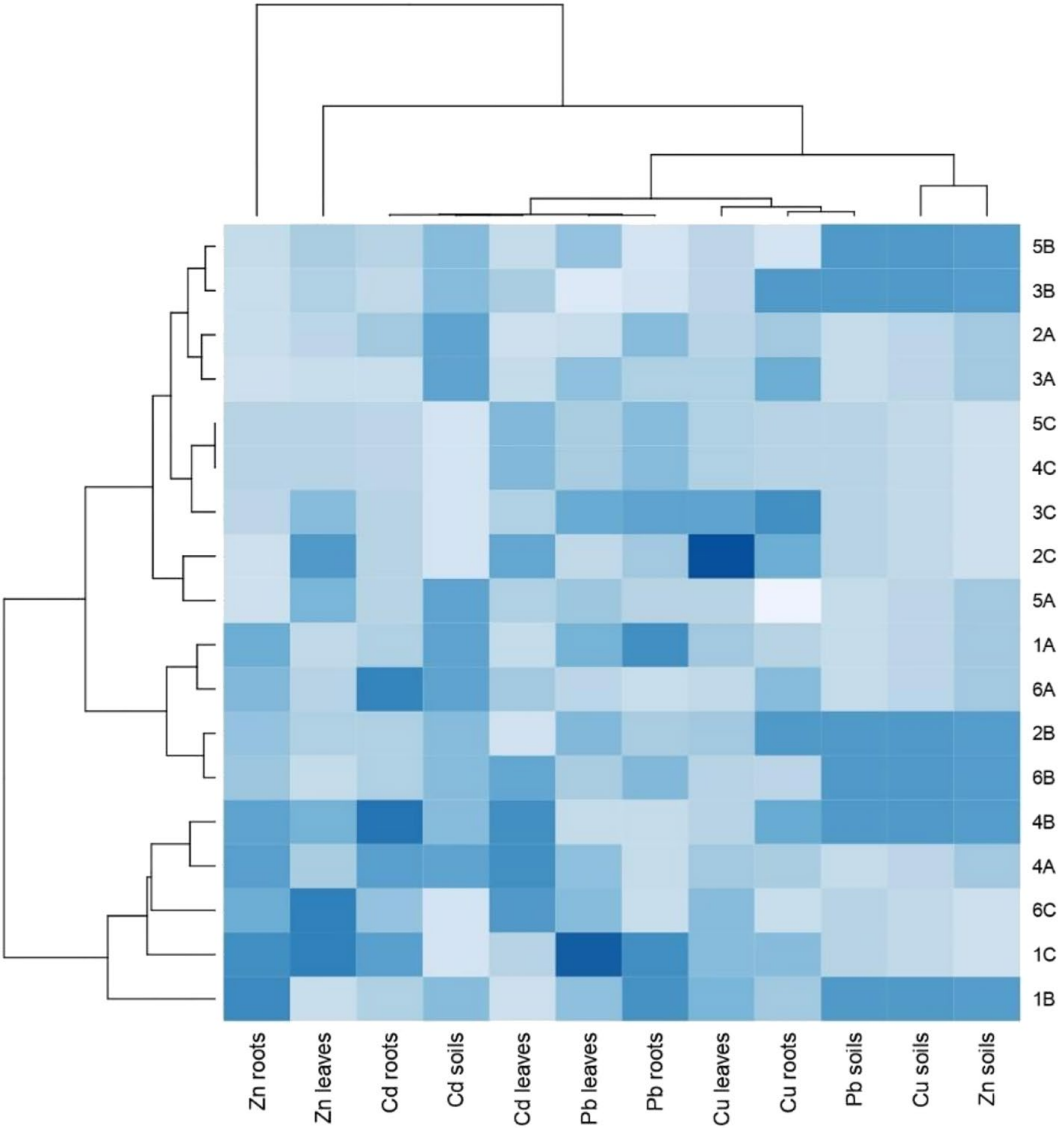
Heatmap and cluster analysis of heavy metal contents in soils, roots and leaves in examined samples at all research sites (abbreviations see “Materials and methods”).

Based on the cluster analysis with the procedure for grouping objects and features (Fig. 1), taking into account all detected heavy metals, it can be found that there were differences between samples from sites A, B and C. The soil most contaminated by Pb, Cu and Zn and Cd was from site B—the Botanical Garden. Two groups of samples were formed. The first consisted of 1B, 1C, 4A and 4B, and 6C. The second consisted of four subgroups: the first subgroup included samples 6B, 2B, 6A and 1A; the second 5A, 2C; the third 3C, 4C, 5C; and the fourth 3A, 2A, 3B, 5B. Regarding the values of heavy metals in roots of detected species, the highest concentration of Zn in roots was detected in *L. multiflorum* (1B, 1C, 1A), *A. rosea* (4A, 4B) and in *P. lanceolata* (6A, 6C); the highest values of Cd in roots was detected in *A. rosea* (4A, 4B), *L. multiflorum* (1C), and *P. lanceolata* (6A) samples; the highest concentration of Pb in roots was noted in *L. multiflorum* (1B, 1C, 1A), *P. lanceolata* (6B), *R. acetosa* (3C), *A. rosea* (4C), *A. retroflexus* (5C) and in *T. pratense* (2A). In sequence, taking into consideration Cu in roots, we noted the highest values in *R. acetosa* (3C, 3A and 3B), *T. pratense* (2B, 2C), *A. rosea* (4B) and *P. lanceolata* (6A). Regarding the values of heavy metals in leaves of detected species, the highest concentrations of Zn were noted in *L. multiflorum* (1C), *P. lanceolata* (6C and 3C), *A. rosea* (4B), *A. retroflexus* (5A), and *T. pratense* (2C), while the highest amounts of Cd were detected in leaves of the following samples: *P. lanceolata* (6B, 6C), *A. rosea* (4A, 4B, 4C), *T. pratense* (2C), and *A. retroflexus* (5C). Relatively high values of Pb in leaves were detected in *L. multiflorum* (1C, 1A), *T. pratense* (2B), *R. acetosa* (3C, 3A) and *A. retroflexus* (5B), and finally, the highest concentrations of Cu in leaves were detected in the following samples: *T. pratense* (2C) and *R. acetosa* in (3C).

### Bioconcentration and translocation factor

The bioconcentration factors (BCF) exceeded a value of 1 for Zn and Cd in all plant species. The highest values of Zn BCF were recorded in *L. multiflorum* (1C: BCF_Zn_ = 6.62), in *A. rosea* (4A: BCF_Zn_ = 5.17) and in *P. lanceolata* (6C: BCF_Zn_ = 4.70); however, the highest Cd BCF values were found in *A. rosea* (4B: BCF_Cd_ = 8.51), in *P. lanceolata* (6A: BCF_Cd_ = 6.94) and in 1C *L. multiflorum* (BCF_Cd_ = 6.29). Cu and Pb bioconcentration was not as effective as the first two elements, but it is worth mentioning that Cu bioconcentration factors of all detected samples exceed bioconcentration of Pb. Taking into account translocation of detected HMs, the highest Cu TF value was detected in sample 2C of *T. pratense* (TF_Cu_ = 2.55), in sample 6C of *P. lanceolata* (TF_Cu_ = 1.55), in 5A sample *A. retroflexus* (TF_Cu_ = 1.50), and in all samples of *Lolium multiflorum* (TF_Cu_ = 1.31–1.08). The Zn translocation factor was the highest in *T. pratense* 2C (TF_Zn_ = 2.67), in all samples of *A. retroflexus* (TF_Zn_ = 2.23–1.05), and in *R. acetosa* (TF_Zn_ = 1.28–1.11). The highest Cd translocation factor was detected in 2C *T. pratense* (TF_Cd_ = 1.97), followed by *P. lanceolata* (TF_Cd_ = 1.51–1.24), and in *R. acetosa* (TF_Cd_ = 1.44–1.42). Regarding the Pb translocation factor, the highest values were detected in *A. retroflexus* (TF_Pb_ = 3.09), in *P. lanceolata* in 6B (TF_Pb_ = 2.25), and in sample 4A of *A. rosea* (TF_Pb_ = 1.90) (Table 1).

**Table 1:** The bioconcentration (BCF) and translocation factor (TF) of Cu Zn, Cd and Pb in plant species from research sites (abbreviations see “Materials and methods”).

Plants		Bioconcentration factor (BCF)				Translocation factor (TF)			
		Cu	Zn	Cd	Pb	Cu	Zn	Cd	Pb
*Lolium multiflorum*	1A	0.48	**4.24**	**1.71**	0.18	**1.08**	0.30	0.45	0.58
	1B	0.35	**5.92**	**1.76**	0.16	**1.31**	0.17	0.25	0.50
	1C	0.64	**6.62**	**6.29**	0.18	**1.08**	0.50	0.23	**1.09**
	Control	0.43	**3.64**	**2.01**	0.11	**1.16**	0.36	0.22	0.73
*Trifolium pratense*	2A	0.53	**1.18**	**1.99**	0.11	0.72	**1.11**	0.24	0.43
	2B	0.51	**2.77**	**1.71**	0.07	0.64	0.47	0.22	**1.19**
	2C	0.71	**1.05**	**1.74**	0.08	**2.55**	**2.67**	**1.97**	0.62
	Control	0.73	**1.41**	**1.66**	0.08	0.99	**1.15**	0.33	0.62
*Rumex accetosa*	3A	0.67	0.87	0.49	0.07	0.62	**1.28**	**1.42**	**1.28**
	3B	0.51	**1.13**	**1.02**	0.03	0.48	**1.15**	**1.44**	0.86
	3C	0.86	**1.84**	**1.71**	0.15	**1.09**	**1.11**	0.92	0.76
	Control	0.98	**1.09**	**1.11**	0.04	**0.75**	**1.05**	1.20	0.96d
*Alcea rosea*	4A	0.52	**5.17**	**4.93**	0.05	0.99	0.29	0.73	**1.90**
	4B	0.45	**4.53**	**8.51**	0.04	0.58	0.42	0.46	**1.06**
	4C	0.51	**2.20**	**1.50**	0.11	0.91	0.69	**1.81**	0.67
	Control	0.80	**3.62**	**2.81**	0.04	0.78	0.54	0.65	**1.09**
*Amaranthus retroflexus*	5A	0.26	0.90	**1.30**	0.07	**1.50**	**2.23**	0.91	**1.16**
	5B	0.24	**1.30**	**1.47**	0.02	0.94	**1.05**	0.57	**3.09**
	5C	0.32	**1.11**	**1.37**	0.04	**1.17**	**1.50**	0.73	**1.71**
	Control	0.52	**1.09**	**1.62**	0.04	**1.01**	**1.73**	0.57	**1.13**
*Plantago lanceolata*	6A	0.61	**3.77**	**6.94**	0.04	0.55	0.37	0.22	**1.35**
	6B	0.31	**2.66**	**1.94**	0.10	0.80	0.41	**1.51**	0.62
	6C	0.44	**4.70**	**3.21**	0.04	**1.55**	0.70	**1.24**	**2.25**
	Control	0.76	**2.36**	**2.46**	0.06	**1.20**	0.53	**1.07**	**1.40**

Where: in BCF the highlighted values mean concentration in roots biomass and in TF the highlighted values means effective metals translocation within the plant.

### Physiological condition of species

After 6 weeks of the experiment, cell membrane stability (MSI) took values from 93.41% in *Alcea rosea* sample 4C to the highest values, more than 98%, in all *Plantago lanceolata* samples, and in samples of *Trifolium pratense*, *Rumex acetosa* and *Amaranthus retroflexus*. Dry mass content was highest (23.11%) in *Amaranthus retroflexus* sample 5A, and the lowest in *R. acetosa* (8.01%) in sample 3C. In all species higher dry mass contents was detected in comparison to control samples. RWC was the highest in *R. acetosa* sample 3A (95.25%) and the lowest in *A. rosea* sample 4C (62.53%). It should be noted that almost all samples were characterized by RWC above 90%. Chlorophyll *a* content ranged from 3.33 in *Amaranthus retroflexus* to 11.02 in *Rumex acetosa*. Chlorophyll *b* content ranged from 4.96 in *Trifolium pratense* to 0.9 in *Amaranthus retroflexus*. Chlorophyll *a* + *b* content ranged from 4.11 in *A. retroflexus* to 15.36 in *R. acetosa.* Chlorophyll *a/b* ratio ranged from 0.27 in *A. retroflexus* to 0.58 in *T. pratense*. Photosynthesis activity (*P*_N_) ranged in detected species from 7.24 in *Rumex acetosa* to 28.28 in the *Trifolium pratense* sample. Stomatal conductance (*g*_s_) ranged from 32.64 in *Amaranthus retroflexus* to 188.86 in *Trifolium pratense*. *C*i intercellular CO_2_ concentration varies from 261.47 in *Amaranthus retroflexus* to 528.20 in *Plantago lanceolata* (Table 2).

**Table 2:** Cell membrane stability—MSI (%), dry mass (%), relative water content—RWC (%), chlorophyll [Chl *a*, Chl *b*, Chl *a* + *b*, ratio *b/a*] (mg g^−1^), and photosynthetic activities—net photosynthetic rate—*P*_N_ (µmol CO_2_ m^−2^ s^−1^), stomatal conductance—*g*_s_ (µmol CO_2_ m^−2^ s^−1^) and intercellular CO_2_ concentration—*C*i (*µ*mol CO_2_ m^−2^ s^−1^) detected for all samples (abbreviations see “Materials and methods”).

Plants		MSI	Dray mass	RWC	Chl *a*	Chl *b*	Ch *a* + *b*	Ratio *b/a*	P_*N*_	*g*_s_	*C*i
*Lolium multiflorum*	1A	97.99	20.00	94.30	5.84	1.91	7.74	0.33	10.67	85.84	471.82
	1B	94.30	14.85	91.89	7.33	2.39	9.58	0.32	16.43	123.64	436.27
	1C	96.82	16.19	64.57	6.00	1.64	7.60	0.26	15.18	115.29	507.37
	Control	98.10	10.48	96.51	9.41	2.98	12.35	0.32	10.77	70.05	233.93
*Trifolium pratense*	2A	96.64	19.97	93.56	7.95	4.00	12.80	0.50	20.71	108.62	331.63
	2B	98.27	20.50	93.36	5.78	2.01	7.74	0.35	16.30	188.86	518.13
	2C	96.76	16.93	90.74	8.79	4.96	14.84	0.58	28.28	172.52	459.57
	Control	95.86	11.51	93.77	17.85	5.30	22.89	0.30	9.10	38.43	254.63
*Rumex accetosa*	3A	97.66	12.73	95.25	5.15	2.01	7.14	0.39	7.24	38.28	357.50
	3B	98.83	14.30	90.90	6.55	2.51	9.04	0.38	7.49	75.96	506.33
	3C	97.03	8.01	92.08	11.02	4.33	15.36	0.39	13.47	99.45	517.57
	Control	94.95	11.86	89.68	7.06	2.19	9.16	0.31	18.27	116.93	284.43
*Alcea rosea*	4A	95.02	21.78	78.23	4.51	1.52	6.02	0.34	8.66	44.48	308.00
	4B	94.37	19.09	78.78	6.80	2.28	9.03	0.34	12.29	84.37	430.90
	4C	93.41	22.34	62.53	6.02	2.20	8.34	0.37	11.65	58.14	392.00
	Control	95.03	13.19	80.94	14.54	16.01	30.11	0.75	9.75	57.67	232.53
*Amaranthus retroflexus*	5A	97.44	23.11	91.75	3.76	1.13	4.81	0.30	18.21	81.08	261.47
	5B	98.21	22.07	95.38	3.33	0.90	4.11	0.27	15.42	77.16	341.03
	5C	97.38	14.19	94.11	10.27	4.06	14.84	0.39	8.45	32.64	309.50
	Control	94.13	9.26	91.04	12.83	3.21	15.73	0.25	6.70	57.67	232.53
*Plantago lanceolata*	6A	98.23	11.62	92.23	8.34	3.15	11.28	0.38	12.37	86.46	391.97
	6B	98.76	16.77	93.62	3.93	1.28	5.11	0.33	16.87	128.84	446.53
	6C	98.29	10.20	93.92	8.58	3.01	11.48	0.35	15.60	131.24	528.20
	Control	98.78	6.88	92.29	13.88	4.41	18.15	0.32	10.49	88.90	555.80

**Figure 2:**
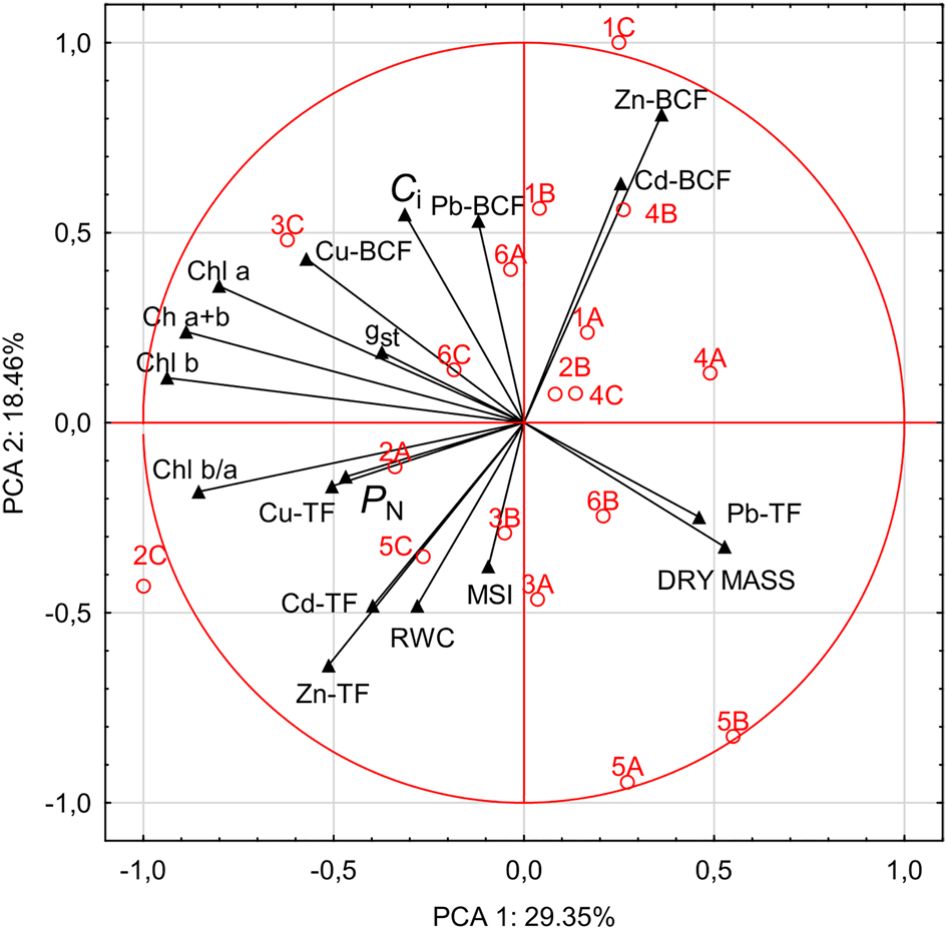
Principal component analysis of heavy metal concentrations, bioconcentration factor (BCF) and translocation factor (TF) in examined samples (abbreviations see “Materials and methods”) in relation to photosynthesis activity parameters: net photosynthetic rate *P*_N_, stomatal conductance g_s_ and intercellular CO_2_ concentration and (*C*i), chlorophyll parameters and dry mass, relative water content (RWC) and cell membrane stability (MSI).

The graphical representation of the results by analysis of the first two principal components for heavy metal accumulation in leaves, roots and photosynthesis activity parameters in all samples explained more than 47.81% of total variability (Fig. 2). A positive relationship was found between Zn-BCF, Cd-BCF in *L. multiflorum* samples (1A, 1B, 1C), *T. pratense* (2B) samples and in *A. rosea* (4B, 4C) samples. We also found a positive relationship between Pb TF and dry mass content in *P. lanceolata* (6B). Another large group consists of correlated Zn TF, Cu-TF, Cd TF with relative water content (RWC), cell membrane stability (MSI), chlorophyll b/a coefficient and net photosynthetic rate – *P*_N._ of *T. pratense* (2A, 2C), *R. acetosa* (3A, 3B) and *A. retroflexus* (5C) samples. The last group was composed of Pb-BCF, Cu-BF correlated with chlorophyll content stomatal conductance (g_s_) and intercellular CO_2_ concentration (*C*i) of *R. acetosa* (3C) and *P. lanceolata* (6A, 6C) samples.

The profiles of changes and the level of hydrogen peroxide values in all species were similar in roots and in leaves, with the highest amount of H_2_O_2_ ≈ 6 (nmol H_2_O_2_ × min^−1^ × mg protein^−1^) in roots of *R. acetosa* and *A. rosea* (4A). The highest amount of H_2_O_2_ ≈ 5 (nmol H_2_O_2_ × min^−1^ × mg protein^−1^) in leaves was detected in all samples of *T. pratense*, *A. retroflexus* and in *A. rosea* sample 4A (Fig. S1).

The profiles of changes and the level of CAT activity were similar in roots in leaves of *L. multiflorum*, *R. acetosa* and *A. retroflexus*. The highest activity of CAT ≈ 1.5 (nmol H_2_O_2_ × min^−1^ × mg protein^−1^) in roots and CAT ≈ 0.9 (nmol H_2_O_2_ × min^−1^ × mg protein^−1^) in leaves was noted in *L. multiflorum*. It is worth noting that in *A. retroflexus* and in *P. lanceolata* high activity of CAT ≈ 0.9 (nmol H_2_O_2_ × min^−1^ × mg protein^−1^) was noted in roots. In leaves the highest activity was noted in *L. multiflorum* CAT ≈ 0.9 (nmol H_2_O_2_ × min^−1^ × mg protein^−1^) and in A. retroflexus CAT ≈ 0.55 (nmol H_2_O_2_ × min^−1^ × mg protein^−1^) (Fig. S2).

APOX activities were generally higher in roots than in leaves. In roots, the highest activity (APOX ≈ 0.065) was noted in *L. multiflorum* and in *P. lanceolata*. In leaves APOX activity in all samples was high, with the highest activity of APOX ≈ 0.03 in *L. multiflorum* and in the *A. rosea* sample (Fig. S3).

The profiles of changes and the level of MDA activity were higher in roots than in leaves of *T. pratense*, *R. acetosa*, *A. rosea* and *A. retroflexus*. The highest level of MDA ≈ 12.0 in roots and in leaves (MDA ≈ 7.0) was detected in *A. rosea* sample 4A (Fig. S4).

**Figure 3:**
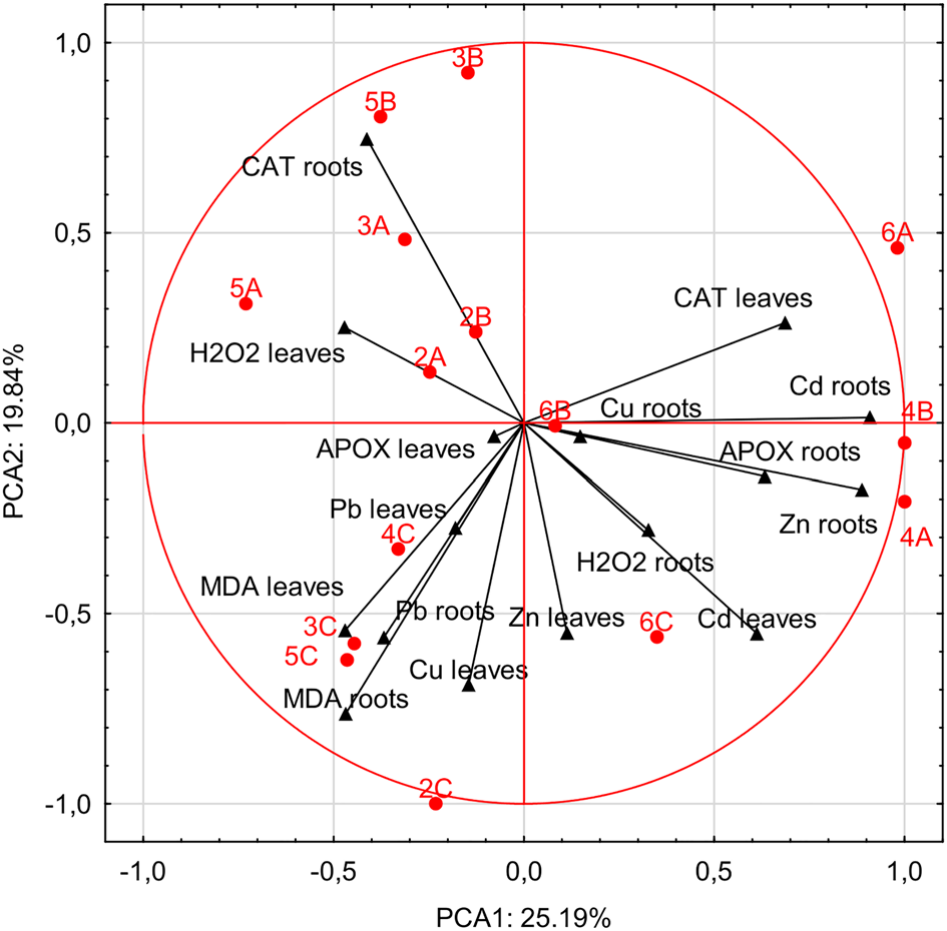
Principal component analysis of heavy metal concentration in examined samples (abbreviations see “Materials and methods”) in relation to hydrogen peroxide—H_2_O_2_, APOX, CAT, MDA activities.

The graphical representation of the results by the analysis of the first two principal components for heavy metal accumulation in leaves, roots and photosynthesis activity parameters in all samples explained more than 45% of the total variability (Fig. 3). A positive relationship was found between CAT activity in leaves and Cd and Cu in roots of *Plantago lanceolata* samples 6A and 6B and *Alcea rosea* sample 4B. APOX activities in roots were related to Zn in roots in *Alcea rosea* sample 4A. There was a positive relationship between hydrogen peroxide amount and Cu and Zn in leaves of *Plantago lanceolata* sample 6C. The next group consisted of APOX in leaves, MDA content in roots and the level of Pb in roots and leaves, the level of Cu in leaves in *A. rosea* 4C, *R. acetosa* 3C, *A.* retroflexus 5C and *T. pratense* 2C samples. Finally, there was a relationship between hydrogen peroxide in leaves, CAT activity in roots in *A. retroflexus* 5A and B, *R. acetosa* 3A and *T. pratense* 2A and B samples.

**Figure 4:**
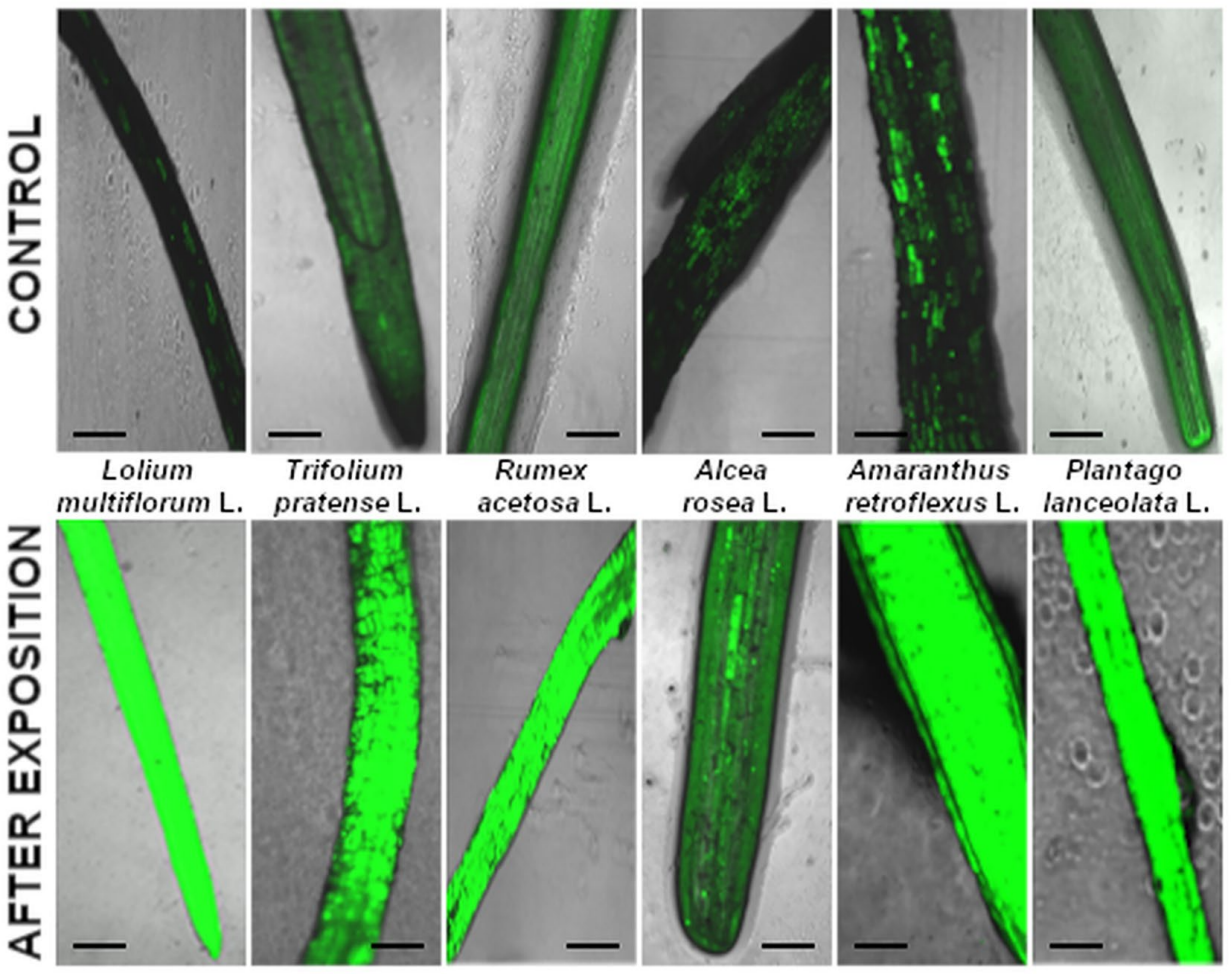
Fluorescent images show H_2_O_2_ production in leaves of examined species after exposure, (abbreviations see “Materials and methods”). The bar indicates 1 µm.

The most intensive fluorescence DHE, indicating the presence of H_2_O_2_ in leaves, was observed in the *T. pratense* (2A), *Rumex acetosa* (3A), A. rosea (4A) and *P. lanceolata* (6A) samples (Fig. 4).

## Discussion

This comparative study of different weed species has shown their potential for accumulation of HMs. *Lolium multiflorum* var. Ponto was used as a known variety cumulating HMs^19, 25, 26^, but in our study it was found that this variety showed similar possibilities of HM accumulation to the weed species selected for the experiment. Almost all the studied species showed significant potential for Zn and Cd accumulation compared to Cu and Pb. The efficiency of accumulation was expressed as the BCF factor, and the metal displacement efficiency as the translocation factor (TF). For a hyperaccumulator plant, both of these factors should be greater than unity^27^. Taking into account these two factors, the most effective bioindicator can be selected. Analyzing cadmium, which is the metal that is the most toxic to plants and animals^28^, the highest content of Cd was found in the roots of *A. rosea* and *P. lanceolata*. Very high bioconcentration coefficients calculated for these two species confirmed the potential for this element to be concentrated in these species’ tissues. The usefulness of *A. rosea* as a hyperaccumulator of Cd was noted previously by Liu et al.^29^ and Ubeynarayana et al.^30^. Zn was also effectively accumulated mainly in leaves by *A. rosea* and *P. lanceolata*. However, it is worth noting that Zn was well accumulated by all analyzed species. This ability was also confirmed by bioconcentration factors determined for this species. Zinc as an essential micronutrient in plant nutrition is naturally taken up by plants, and the efficiency of uptake depends on soil pH and phosphorus levels. In the countries of the European Union, soil is polluted with zinc, due to the use of sewage sludge for fertilization purposes or composts made from them^31^. The amounts of Zn in unpolluted soils typically are lower than 125 ppm (125 mg kg^−1^) and in plants growing in these soils this metal concentration varies between 0.02 and 0.04 mg g^−1^ (20–40 mg kg^−1^) dry weight^32^. In our study, the highest concentration of Zn (≈ 29 mg/kg) was detected in Botanical Garden soil (B), which could be the consequence of use of fertilizers in this area. However, according to Polish regulations on the permissible content of substances causing risk to human health and the environment, the obtained results do not exceed the standards for soil at urban areas (Cu = 200 mg kg^−1^, Zn = 500 mg kg^−1^, Cd = 2 mg kg^−1^, Pb = 200 mg kg^−1^)^33^. Duan et al.^34^ noted that Zn was accumulated more in roots than in leaves of *A. rosea*, which was confirmed in our study; the most effective accumulation of Zn was also detected in *A. rosea* as well as in *P. lanceolata* and *L. multiflorum* var. Ponto. We confirmed earlier information about mobility of Zn in plant tissues. The best Zn-TF transport efficiency was detected in *R. acetosa*.

Longnecker et al.^35^ reported that in plants tolerant of toxic levels of Zn, accumulation was observed in the root cortex and in their leaves. We detected that cadmium was effectively accumulated in the roots, but also efficiently transported to the leaves of *A. rosea*, *P. lanceolata*, *T. pratense* and *R. acetosa* samples, as evidenced by high translocation factor (TF) values. If we take into account Zn and Cd together, we should remember about the interaction between Zn and Cd, which consists in the mutual inhibition of the accumulation and sometimes translocation of elements in the plant^36^.Recent research showed that the use of excess Zn, along with exposure to Cd under hydroponic conditions, mitigated Cd toxicity in plants by increasing all phenols and chlorophylls in the leaves, thereby mitigating the adverse effects of Cd on photosynthetic function and oxygen secretion activity^37^. The authors suggested that these mechanisms are involved in Zn detoxification and protection against Cd-induced structural and functional damage of the photosynthetic membranes.

Pb accumulation was not so effective as mentioned earlier for Zn and Cd, but it is worth pointing out that a relatively high concentration was found in leaves of all examined species, the highest in *L. multiflorum* and *R. acetosa*. Barrutia et al.^38^ established that *R. acetosa* had the potential for bioconcentration of Pb (and also Zn and Cd), from highly contaminated mine soil. However, our research did not confirm the ability to hyperaccumulate this element, which could be due to there being a relatively small amount in the soil of the studied sites. Lead was efficiently transported to the above part of the plant in selected samples of all examined species, especially in *A. retroflexus* and also in *A. rosea* and *P. lanceolata*. Mobility of this ion in *Amaranthus spinosus* was also confirmed by Yingping et al.^39^. In *Limbarda crithmoides* and *Helianthus annuus* Pb was not efficiently transported from roots to the leaves^40^ and it was only accumulated in roots.

Taking into consideration Cu, we noted that *L. multiflorum*, *T. pratense*, *R. acetosa*, *A. retroflexus* and *P. lanceolata* showed the ability to accumulate copper and its quick transport from the roots to the above-ground parts of the plant. Malizia et al.^1^ confirmed that *T. pratense* has the potential for copper bioconcentration.

Our results also demonstrate a synergistic interaction between Cu, Cd and Zn during translocation of these elements in shoots of *Trifolium pratense* and *Amaranthus retroflexus* (Fig. 2). Other researchers have also noted that *Amaranthus retroflexus* accumulates metals such as Cd, Ni, Pb and Cu in the aboveground parts^41^. In *Rumex acetosa* an interaction between translocation of Cd and Zn (Fig. 2) was found, whereas other authors^42^ reported that Zn induced a decrease in Cd uptake and a simultaneous increase in Zn accumulation in tomato plants. This suggested strong competition between Zn and Cd for the same membrane transporters.

In the presence of heavy metal ions, the plants in our study did not exhibit characteristic symptoms of their toxic effect on physiological activity. Dry mass, RWC, net photosynthetic rate *P*_N_, stomatal conductance g_s_ and intercellular CO_2_ concentration *C*i in plants after exposure were higher than in control species; only in one species—*R. acetosa—*did we detect a decrease in these parameters compared to the control (Table 2). This may be due to relatively small amounts of heavy metals in the environment and relatively short exposure. The metal hyperaccumulating plants have an ability to accumulate a relatively high level of HMs in their plant tissues, and they have developed a number of detoxification mechanisms for acclimation and tolerance of metals. The mechanism of Cd tolerance has been extensively studied in many species^43^. Our research showed that species that accumulated Cd effectively triggered detoxification mechanisms to protect the function of the photosynthetic apparatus against Cd stress^44^. Other authors observed a decrease in all photosynthesis parameters in *Amaranthus spinosus*^39^ and delayed chlorophyll fluorescence in *Lemna minor*^45^.

Contamination of plant tissues by heavy metals leads to the formation of ROS such as hydrogen peroxide (H_2_O_2_)^12^. We observed an increase in the level of ROS compared to control plants in all plants after exposures. We observed higher levels of H_2_O_2_ in all plant organs of *T. pratense*, *R. acetosa*, *A. rosea* and *P. lanceolata*. The increase in ROS production in plants was associated with an increase in the activity of antioxidant enzymes.

All examined species were characterized by the potential for accumulation of heavy metals. However, an effective bioaccumulation process will depend on active detoxifying enzymes and the regulation of primary defense enzymes. We always observed the induction of antioxidant enzyme activity in roots and leaves of plants, although there were no significant differences between the researched plants. Heavy metals modify membrane properties by interacting with functional groups of membrane proteins and lipids. As a lipid peroxidation marker^46^, the measurement of malondialdehyde (MDA) content is used. In our research, MDA level activity after 2 weeks was higher than after 6 weeks of exposure. The increase in MDA was induced by both essential metals such as Zn and non-essential metals such as Pb. It was detected that a higher MDA level in roots and leaves in *Lolium multiflorum*, *Trifolium pratense* and *Amaranthus retroflexus* was correlated with high amounts of Pb in roots (Fig.). Lukatkin et al.^22^ reported that MDA levels in roots and leaves of *Amaranthus retroflexus* were correlated with high levels of Zn. Ascorbate peroxidase (APOX) isoforms play important and direct roles as protective elements against adverse environmental conditions^42^. The decrease in membrane lipid peroxidation observed after 6 weeks may be due to activation of the ROS-inactivating antioxidant system. In our study APOX activities were generally higher in leaves than in roots in all species. Activity of APOX was definitely lower than catalase, especially in the above-ground parts, which means that this enzyme complements CAT catalytic activity. APOX may be responsible for controlling the levels of H_2_O_2_ as signal molecules, and the CAT function is to remove large amounts of oxygen during oxidative stress^12^. The profiles of changes and the activity level of CAT were different between leaves and roots, but it is worth noting that in all sites with exposure of *A. retroflexus* higher activity values of CAT were noted. Mohamed et al.^47^ showed in *Brassica juncea* that the higher activity of antioxidant enzymes offers greater detoxification efficiency, which provides better plant resistance against trace metal-induced oxidative stress.

The results show the high accumulation potential of these species and their adequate physiological response to stress. In order to detect environmental pollution, data from all tested species should be obtained, and such a procedure will more effectively determine the levels of risk for heavy metals environment contamination.

## Conclusions

Based on the obtained results, it can be concluded that all species showed varied but generally great potential for high accumulation of detected trace elements. The concentrations of all elements in plant tissues were dependent on species, organ (root vs. shoot), and species-organ interactions. The physiological response of the studied species to stress was correlated with the high content of the tested metals in the tissues. Plants exposed in different study sites showed different concentrations of trace elements in their tissues. Due to the varying degree of the tested species ability to accumulate trace elements, in order to estimate the degree of environmental pollution by these compounds, we recommend the simultaneous use of all species of weed which were tested in this work.

In the future, finding a bioindicator among weeds that would have a whole set of excellent bioindicating features, would provide a simple and cheap early warning system against the negative effects of changes in the ecosystem.

## Materials and methods

In order to evaluate the bioaccumulation ability of Cd, Pb, Cu and Zn by 6 selected plant species, an experiment was organized. The contents of these elements, their bioconcentration and translocation in plants and soil were also determined, as well as the condition of the plants was evaluated. In addition, the results were analyzed using statistical analysis.

### Materials

Species for the study were selected due to their common, wide range of occurrence, generally throughout Europe. For this investigation we selected species often found as weeds and as ornamental species in urbanized areas:

*Lolium multiflorum* L. (no. 1) is native to all Europe (except Finland), Western, Southern and Central Asia (except Uzbekistan), as well as Northern Africa. It was introduced to the Americas, South and East Africa, Australia and East Asia (Hultén and Fries, 1986; POWO, 2019). For our purposes we used the *Lolium multiflorum* variety Ponto, obtained from Norddeutsche Pflanzenzucht Hans-Georg Lembke KG (Germany). It displayed phytoremediation potential for heavy metals^48^.

*Trifolium pratense* L. (no. 2) commonly known as red clover. It is a species native to: Europe, South, West and Middle Asia as well as North-West Africa. Introduced and widespread in all continents except Antarctica^49, 50^. The seeds were collected by the authors from Wielkopolska rural areas;

*Rumex acetosa* L. (no. 3), also known as common sorrel, is a herbaceous plant native to Europe, Asia, and North Africa (Morocco), and now it has spread to all continents except Australia and Antarctica^49, 50^. The seeds were collected by the authors from Wielkopolska rural areas;

*Alcea rosea* L. (no. 4), the common hollyhock; it was imported into Europe from southwestern Asia as an ornamental plant species before the fifteenth century. Since then, it is common ornamental plants in cities and widespread in the Americas, North Africa, and South Asia as a wilderness species^49, 50^. In the present experiment we used *Alcea rosea* L. The seeds were collected by the authors from Wielkopolska rural areas;

*Amaranthus retroflexus* L. (no. 5), the red-root amaranth, is now found nearly worldwide. It is a species native to Mexico. It was introduced into all continents except Antarctica^49, 50^. The seeds were collected in the Poznań agglomeration;

*Plantago lanceolata* L. (no. 6), ribwort plantain; this remarkably widespread species is native to all Europe, North Africa and West, South and Middle Asia but has been introduced extremely widely elsewhere and now occurs e.g. in both Americas, Australia, New Zealand, Japan and in South and East Africa, where it thrives at high altitude^49, 50^. The seeds were collected by the authors from Wielkopolska rural areas.

We confirm that all methods including collection of plant material, were carried out in accordance with relevant guidelines and regulations.

### Organization of the experiment

**Figure 5:**
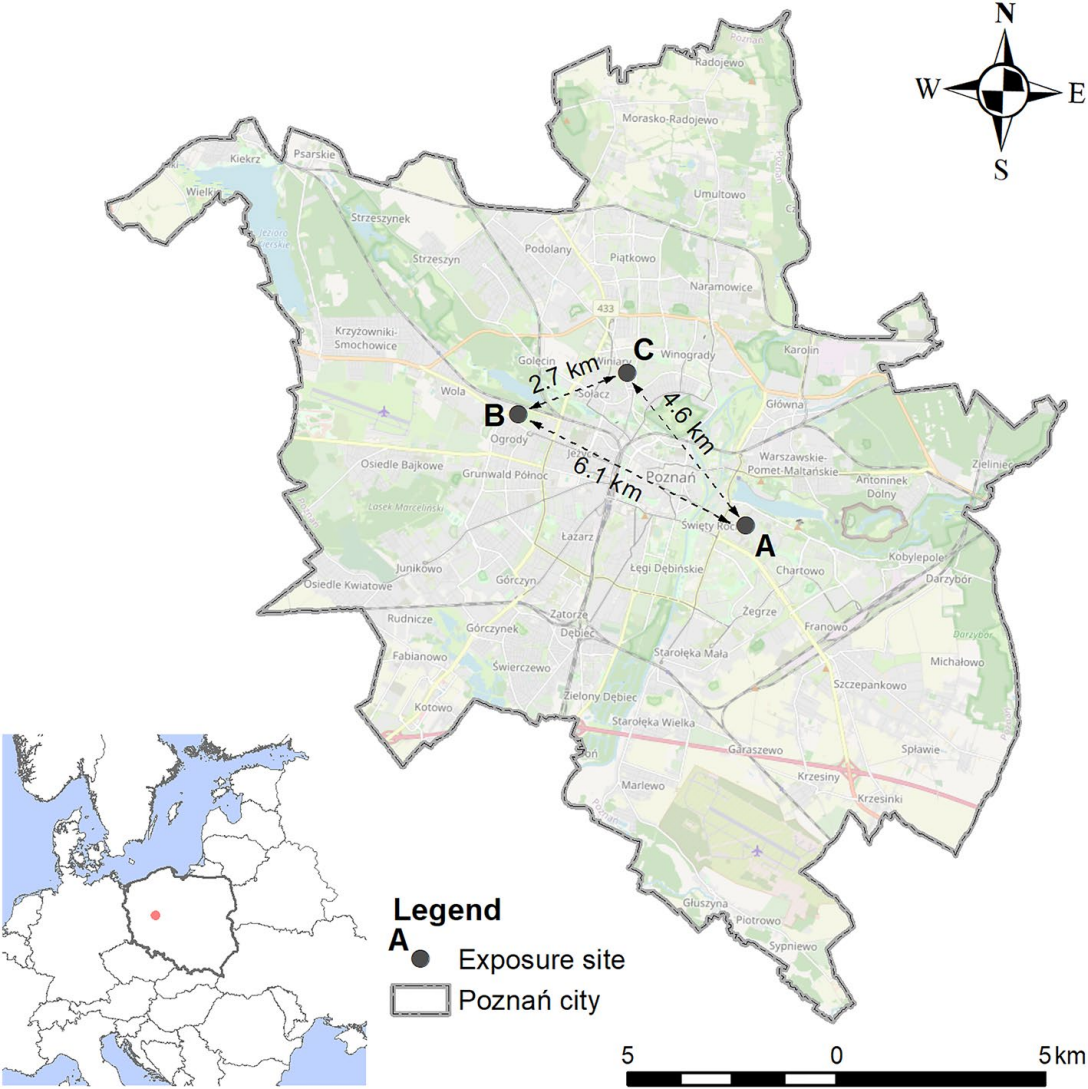
Localization of exposure sites in Poznań city with information about distance between them (source: own study based on data from National Geodetic and Cartographic Resource and © OpenStreetMap and contributors CC-BY-SA).

The experiment was carried out during the growing season in 2021. The experiment started in April with planting the seeds in the control greenhouse conditions (temperature 16–18 °C, no artificial light). 5 L pots with a standard mixture of peat and sand were used (pH 6.8, N: 230 mg L^−1^, P: 180 mg L^−1^, K: 350 mg L^−1^, Mg: 150 mg L^−1^). The content of the tested elements in potting soil at the beginning of the experiment was 4.151 ± 0.032 mg kg^−1^ for Cu, 15.03 ± 0.34 mg kg^−1^ for Zn 0.091 ± 0.004 mg kg^−1^ for Cd, and 4.302 ± 0.052 mg kg^−1^ for Pb. Seeds of each species were sown in individual pots in equal amounts. After germination, ten of the most vital and largest seedlings were left in the pot. During germination and cultivation in the greenhouse, deionized water was used for plant irrigation. After 60 days, the plants were taken to exposure sites with various environmental conditions. The three exposure sites were selected for these investigations, located in Poznań city. The first site (site A) was located in a residential area located on the right bank of the Warta river (N: 52°23′53′′; E: 16°57′36′′), in the eastern part of the Poznań city (Fig. 5). High-density built-up areas (multi-family housing) dominated in the surrounding area of this research site. The second exposure site (site B) was located in the Botanical Garden of the Adam Mickiewicz University in Poznań on the left bank of the Warta river (N: 52°25′14′′; E: 16°52′39′′), in the western part of the city. The immediate surroundings were green areas, whereas in the further surroundings of exposure site C there was a low-density built-up area (single-family housing) and main road (N: 52°25′50′′; E: 16°54′58′′). At each site, three pots with plants of a given species were placed (in total, there were 18 pots per site). The exposure of samples lasted 6 weeks (from June 1 to July 16, 2021). During the exposure, the plants were watered with distilled water and protected from the direct sun (shadowed) by naturally occurring higher vegetation, without negative effect for air flow. Air pollution at research sites during exposition, were provided by the General Directorate for Environmental Protection (Table S3).

### Heavy metal analysis

#### Preparation samples

In the laboratory, the plant samples were first purified with deionized water using Milli-Q Advantage A10 Water Purification Systems, Merck Millipore (Merck, Darmstadt, Germany), and separated into leaves and roots. The soil samples from all pots were sieved (2 mm). To achieve constant dry weight, the plant and soil samples were dried at 40 ± 3 °C in an electric oven (FD115, Binder, Germany). Digestion of the powdered samples of plant (homogenous samples from each pot) and soil were carried out in the CEM Mars 5 Xpress microwave mineralization system (CEM, USA). From each plant, 0.3000 ± 0.0001 g of leaves or roots were placed in a Teflon vessel with 8 mL of concentrated (65%) HNO_3_ (analytical purity, Merck, Darmstadt, Germany) and 1 mL of H_2_O_2_ (Merck, Darmstadt, Germany). The program of digestion included the following stages—first stage: temperature to 80 °C, 10 min, power 600 W; second stage: temperature 140 °C, 12 min, power 1200 W; third stage: temperature 185 °C, 15 min, power 1200 W. After the digestion steps using Qualitative Filter Papers (Grade 595: 4–7 μm Whatman, GB), the solutions were filtered, placed in flasks and made up to a final volume of 15 mL with deionized water. The analysis of the element's concentration in the soil was performed in accordance with the PN-EN 16174 standards. Procedural blanks and reference materials were carried out in the same way as the samples in each digestion run.

#### Analytical procedure

Elemental analysis of Cu, Zn, Cd and Pb was carried out using an inductively coupled plasma mass spectrometer (ICP-MS 7100 × Agilent, Santa Clara, CA, USA) equipped with an octopole reaction system (ORS), MicroMist concentric nebulizer, quartz Scott double pass spray chamber, Ni cones, and a quadrupole mass spectrometer. The instrumental parameters were optimized using the Tuning Solution (Agilent). The typical instrument operating conditions for ICP-MS spectrometers were as follows: 1550 W for RF power, 15 L min^−1^ for plasma gas flow rate, 0.98 L min^−1^ for nebulizer gas flow rate, 0.9 L min^-1^ for auxiliary gas flow rate. For the reduction of spectral interferences, helium mode was used. The non-spectral and matrix interferences were reduced by diluting the samples and using an internal standard solution containing 10 µg L^−1^ Rh introduced in parallel with all analyzed solutions. High purity argon (99.999%) was used as a nebulizer, auxiliary, and plasma gas for the ICP-MS (Messer, Chorzów, Poland). Calibration solutions were prepared by appropriate dilution of 10 mg L^−1^ of multielemental stock solution in 5% HNO_3_ (Multi-Element Calibration Standard 3, PerkinElmer, MA, USA). The calibration curves were constructed in the concentration ranges: 0.05–50 µg L^−1^ for Cd and Pb and 0.1–200.0 µg L^−1^ for Cu and Zn.

#### Quality assurance

To evaluate trueness and establish the traceability of the measurement result, certified reference materials (CRM) were used: NIST SRM 1570a Trace Elements in Spinach Leaves (USA), NIST SRM 2711a Montana Soil. The validation parameters linearity, precision, limits of detection (LOD) and trueness were evaluated. The linearity of the calibration curve was calculated as the correlation coefficient (R), the value of which is greater than 0.9996 for all analytes. The LOD for determined elements were calculated according to LOD = 3.3 S/b, where S means standard deviation of the results obtained for the blank samples and b is the sensitivity (n = 5). The LOD values were as follows: Cd 0.007 µg g^−1^, Cu 0.036 µg g^−1^, Pb 0.008 µg g^−1^ and Zn 0.092 µg g^−1^. Precision values were calculated as the coefficient of variation (CV) (%) ranging from 0.8 to 2.3% for all elements. Trueness was evaluated by applying the certified reference materials and expressed as recovery (%). Recovery values ranged from 97 to 102% for plants and from 93 to 98% for soil, respectively. The results of Student’s t-test also confirmed that there were no significant differences between the measured concentration ± SD and the certified concentration ± standard uncertainty.

#### Accumulation and translocation factor

To estimate the efficiency of heavy metals’ phytoextraction by the studied plant species from three research sites, two factors were calculated: bioconcentration and translocation. The ratio of heavy metal accumulation in root samples to heavy metal accumulation in soil samples was used to calculate bioconcentration factor (BCF) of heavy metals^51^:

$$BCF\, = \,HM \, concentration \, in \, roots \, \left( {{\text{mg kg}}^{ - 1} {\text{DW}}} \right)/ \, HM \, concentration \, in \, soil \, \left( {{\text{mg kg}}^{ - 1} {\text{DW}}} \right).$$

Translocation factor (TF) is efficiency of the heavy metals’ transference to above-ground biomass^52^, with leaves and roots used in the analysis:

$$TF\, = \,HM \, accumulation \, in \, leaves \, \left( {{\text{mg kg}}^{ - 1} {\text{DW}}} \right)/ \, HM \, accumulation \, in \, roots \, \left( {{\text{mg kg}}^{ - 1} {\text{DW}}} \right).$$

### Analysis of physiological conditions of plants

#### Determination of chlorophyll content

To determine chlorophyll content, the experiment was performed in a laboratory, where plants were brought from the three locations of the experiment. In order to avoid chlorophyll degradation, the experiment was carried out in subdued light during analyses and the storage period. Three replicates for each plant sample were made. To determine the content of chlorophyll in plants, first undamaged leaves of the plant were cut and weighed approximately 0.100 g. The weighed samples were cut into smaller pieces and placed in a test tube, then 5 mL of 99.5% DMSO was added, the test tubes were closed with a stopper and they were placed in the refrigerator for 24 h. After 24 h, samples were placed in a water bath at approximately 65 °C for 30–45 min to extract chlorophyll from the leaf blade. Then, the chlorophyll extract was transferred to a 1 cm cuvette and the absorbance was measured on the Hach Lange DR-2800 spectrometer, at three wavelengths: 645 nm, 652 nm, and 663 nm. In parallel time, dry matter determination was carried out for each plant sample. The chlorophyll content in samples was calculated using Arnon’s formula^53^.

#### Cell membrane stability (MSI)

To determine cell membrane stability from each plant, 2 cm^2^ of green leaves (without injury) were cut. Leaves which were cut were rinsed three times with double distilled water and were placed in 25 mL glass beakers, immersed in 10 mL of distilled water then covered with aluminum foil and were put in a refrigerator for 24 h. The same process was repeated after 24 h: the distilled water was removed, leaves were rinsed and were put in the same glass beakers, immersed in 10 mL of double distilled water, covered with aluminum foil and were put in a refrigerator for the next 24 h. After 24 h samples were taken out of the refrigerator and at room temperature their initial conductance was measured. After each measure samples were covered with aluminum foil. Then samples were autoclaved at 0.5 atm, 105 °C for 30 min. After those processes samples were cooled at 25 °C and final conductance was measured. Their respective electric conductivities C1 and C2 were measured by conductivity meters. The membrane stability index was calculated using the equation according to Almeselmani et al.^54^ formula.

#### Relative water content (RWC)

Leaf relative water content (RWC) estimation was done by cutting 3–4 pieces of leaf blade (without injuries). The pieces were weighed and placed in glass beakers. Leaves were pureed in 100 mL of distilled water (completely submerged) and covered with aluminum foil, and they were placed in the refrigerator for 12 h. After 12 h, water was removed from the glass beakers and the samples were dried with tissue paper and then were weighed again. Then after weighing samples, they were placed again in the same beakers and dried at 60 °C for 70 h. After 70 h, the samples were cooled in a desiccator and were weighed again. The RWC value was calculated according to the formula by^55^.

#### Dry matter content in leaves

The drying-weight method was used to determine dry matter content in leaves. For each plant three replications were done. About 1 g of the plant's leaves were cut and were placed into a beaker, which were closed with a watch glass and placed in a dryer for 24 h. Plants were dried at 105 °C until plants had a constant weight. The dry matter content was calculated based on the weight of the plants before and after drying, using formula by Ostrowska et al.^56^.

### Analysis of activity of oxidative stress parameters and level of enzymes of the antioxidative system

#### Photosynthesis

At the beginning, in the middle, and at the end of each exposure series we measured three intensity parameters: net photosynthesis (*P*_N_), intercellular CO_2_ concentration (*C*_i_,) stomatal conductance (*g*_s_). For measurement, matured leaves were selected without mechanical injury. Gas exchange analysis was performed between 09:00 and 15:00 with the aid of the portable photosynthesis system C_i_ 340aa (CID Bioscience Inc., Camas, WA, USA). To ensure similar conditions of measurements in the leaf chamber, stable conditions were provided: CO_2_ inflow concentration (410 µmol (CO_2_) mol^−1^), photosynthetic photon flux density (PPFD) 1000 µmol (photon) m^−2^ s^−1^, a chamber temperature of 25 °C, and relative humidity of 50 ± 3%.

#### Hydrogen peroxide content

The hydrogen peroxide content was determined using the method described by Patterson et al.^57^. The decrease in absorbance was measured at 508 nm using a UV–VIS spectrophotometer (Shimadzu Scientific Instruments, Japan). The reaction mixture contained 50 mM phosphate buffer (pH 8.4) and reagents, 0.6 mM 4-(-2 pyridylazo) resorcinol, and 0.6 mM potassium-titanium oxalate (1:1). The corresponding concentration of H_2_O_2_ was determined against the standard curve of H_2_O_2_.

#### Determination of antioxidative enzyme activities

The activity of catalase (CAT, EC 1.11.1.6) was determined by directly measuring the decomposition of H_2_O_2_ at 240 nm for 3 min as described by Aebi^58^ in a 50 mM phosphate buffer (pH 7.0) containing 5 mM H_2_O_2_ and enzyme extract. CAT activity was determined using the extinction coefficient of 36 mM^−1^ cm^−1^ for H_2_O_2_. The activity of ascorbate peroxidase (APOX, EC 1.11.1.11) was assayed using the method described by Nakano and Asada^59^ by monitoring the rate of ascorbate oxidation at 290 nm (extinction coefficient of 2.9 mM^−1^ cm^−1^) for 3 min. The reaction mixture consisted of 25–50 μL of supernatant, 50 mM phosphate buffer (pH 7.0), 20 μM H_2_O_2_, 0.2 mM ascorbate, and 0.2 mM EDTA.

#### Measurement of lipid peroxidation and protein quantification

Malondialdehyde (MDA) content was determined by reaction with thiobarbituric acid (TBA) as described by Heath and Packer^60^. Total soluble protein contents were determined according to the method of Bradford^61^ using the Bio-Rad assay kit with bovine serum albumin as a calibration standard.

#### In situ detection of hydrogen peroxide

For the in vivo determination of hydrogen peroxide we used a modified version of the method described by Afzal et al.^62^. All plant specimens were submerged for 12 h in 4 µM dichlorodihydrofluorescein diacetate (DCFH-DA) in 5 mM dimethyl sulfoxide (DMSO). After rinsing with 50 mM phosphate buffer (pH 7.4), the roots were observed with a confocal microscope (Zeiss LSM 510, Axiovert 200 M, Jena, Germany) equipped with no. 10 filter sets (excitation 450–490 nm, emission 520 nm or more).

### Statistical analysis

Descriptive statistical analysis was performed to assess the concentrations of heavy metals in examined plant species from different samples and also concentration of defense system and physiological parameters. Statistical analysis was performed for 72 pots (separately for leaves, roots and soil). All samples followed assumptions of distribution normality and homogeneity. Analysis of variance (two-way ANOVA) was used to assess the significance of differences between species and location for all parameters, and finally, the Scheffé test was applied to show the existence of uniform groups of objects (soils, roots and leaves, separately) (α ≤ 0.05). Principal component analysis (PCA) was performed to evaluate associations between elemental contents and different cities and determine interactions between independent variables (relations between elemental contents in species, location and physiological parameters), without any a priori assumptions. Cluster analyses with procedure grouping objects and features, were performed using R platform (R Core 2014), to find similarities between sites, species, and heavy metal accumulations. Data were visualized using heat maps to compare the concentration of a particular group of elements in plants and soils at specific research sites, with two-dimensional variables (research sites, element) represented by colors.

Statistical analyses were carried out using statistical software (Statistica 13.1) and R computer platform (R Core, 2014).

## Supplementary Information

Supplementary Information.

## Data Availability

All data included in this study are available upon request by contact with the corresponding author.

## References

[1] Malizia D, Giuliano A, Ortaggi G, Masotti A (2012). Common plants as alternative analytical tools to monitor heavy metals in soil. Chem. Cent. J..

[2] Koller, M., & Saleh, H. M. Introducing heavy metals. in *Heavy Metals* (eds. El-Din, H., Saleh, M. & Aglan, R. F.). 10.5772/intechopen.74783 (Intech Open, 2018).

[3] Carreras HA, Pignata ML (2002). Biomonitoring of heavy metals and air quality in Cordoba City, Argentina, using transplanted lichens. Environ. Pollut..

[4] Morais, S., Costa, F. G. & Lourdes Pereira, M. Heavy metals and human health. in *Environmental Health—Emerging Issues and Practice* (eds. El-Din, H., Saleh, M. & Aglan, R. F.). 10.5772/intechopen.71185 (IntechOpen, 2012).

[5] Mohanraj RPA, Priscilla AT (2004). Heavy metals in airborne particulate matter of urban Coimbatore. Arch. Environ. Contam. Toxicol..

[6] Nagajyoti PC, Lee KD, Sreekanth TVM (2010). Heavy metals, occurrence and toxicity for plants: A review. Environ. Chem. Lett..

[7] Ackova DG (2018). Heavy metals and their general toxicity for plants. Plant Sci. Today.

[8] Mukesh KR, Kumar P, Singh M, Singh A (2008). Toxic effect of heavy metals in livestock health. Veterin World.

[9] Djingova, R. & Kuleff, I. Instrumental techniques for trace analysis. in *Trace Elements: Their Distribution and Effects in the Environment* (Vernet, J.P. Ed.). Vol. 4. 137–185 (Elsevier, 2000).

[10] Asati A, Pichhode M, Nikhil K (2016). Effect of heavy metals on plants: An overview. Int. J. Appl. Innov. Eng. Manag. (IJAIEM).

[11] Małecka A, Ciszewska L, Staszak A, Ratajczak E (2021). Relationship between mitochondrial changes and seed aging as a limitation of viability for the storage of beech seed (*Fagus sylvatica* L.). PeerJ.

[12] Małecka A, Konkolewska A, Hanć A, Kmita H, Jarmuszkiewicz W (2019). Insight into the phytoremediation capability of *Brassica juncea* (v. Malopolska): Metal accumulation and antioxidant enzyme activity. Int. J. Mol. Sci..

[13] Scandalios JG (2005). Oxidative stress: Molecular perception and transduction of signal triggering antioxidant gene defenses. Braz. J. Med. Biol. Res..

[14] Moustakas M, Hanć A, Dobrikova A, Sperdouli I, Adamakis I-DS (2019). Spatial heterogeneity of cadmium effects on salvia sclarea leaves revealed by chlorophyll fluorescence imaging analysis and laser ablation inductively coupled plasma mass spectrometry. Materials..

[15] Rucandio MI, Petit-Domínguez MD, Fidalgo-Hijano C, García-Giménez R (2011). Biomonitoring of chemical elements in an urban environment using arboreal and bush plant species. Environ. Sci. Pollut. Res..

[16] Giampaoli P, Wannaz E, Tavares A, Domingos M (2016). Suitability of *Tillandsia usneoides* and *Aechmea fasciata* for biomonitoring toxic elements under tropical seasonal climate. Chemiosphere.

[17] Diatta J, Grzebisz W, Apolinarska K (2003). A study of soil pollution by heavy metals in the city of Poznan (Poland) using dandelion (*Taraxacum officinale* Web) as a bioindicator. Electron. J. Pol. Agric. Univ..

[18] Radulescu C, Stihi C, Popescu I, Dulama I, Chelarescu E (2013). Heavy metal accumulation and translocation in different parts of *Brassica oleracea* L. Rom. J. Phys..

[19] Borowiak K, Budka A, Hanć A, Kayzer D, Lisiak M (2018). Relations between photosynthetic pigments macro-elements contents and selected trace elements accumulated in *Lolium multiflorum* L. exposed to ambient air conditions. Acta Biol. Cracoviensia Ser. Bot..

[20] Lisiak-Zielińska M, Borowiak K, Budka A, Kanclerz J, Janicka E (2021). How polluted are cities in central Europe?—Heavy metal contamination in *Taraxacum officinale* and soils collected from different land use areas of three representative cities. Chemosphere.

[21] Nagórska-Socha A, Ptasiński B, Kita A (2013). Heavy metal bioaccumulation and antioxidative responses in *Cardaminopsis arenosa* and *Plantago lanceolata* leaves from metalliferous and non-metalliferous sites: A field study. Ecotoxicology.

[22] Lukatkin AS, Dmitry I, Bashmakov DI, Harbawee WEQ, Teixeira da Silva JA (2021). Assessment of physiological and biochemical responses of *Amaranthus retroflexus* seedlings to the accumulation of heavy metals with regards to phytoremediation potential. Int. J. Phytoremediat..

[23] Ligarda-Samanez CA, Choque-Quispe D, Palomino-Rincón H, Ramos-Pacheco BS, Moscoso-Moscoso E (2022). Modified polymeric biosorbents from *Rumex acetosella* for the removal of heavy metals in wastewater. Polymers.

[24] Kaya I, Gülser F (2018). Original research determining heavy metal contents of Hollyhock (*Alcea rosea* L.) in roadside soils of a Turkish Lake Basin. Pol. J. Environ. Stud..

[25] Klumpp A, Ansel W, Klump G, Breuer J, Vergne P (2009). Airborne trace element pollution in 11 European cities assessed by exposure of standardised ryegrass cultures. Atmos. Environ..

[26] Maciejewska-Malina J, Maciejewska A (2013). Uptake of heavy metals by darnel multifloral (*Lolium multiflorum* Lam.) at diverse soil reaction and organic matter content. Soil Sci. Annu..

[27] Yanqun Z, Yuan L, Jianjun C, Haiyan C, Li Q, Schvartz C (2005). Hyperaccumulation of Pb, Zn and Cd in herbaceous grown on lead–zinc mining area in Yunnan, China. Environ. Int..

[28] Kabata-Pendias A, Pendias H (2001). Trace Elements in Soils and Plants.

[29] Liu J, Zhou Q, Song W, Ting S (2008). Cadmium tolerance and accumulation of *Althaea rosea* Cav. and its potential as a hyperaccumulator under chemical enhancement. Environ. Monit. Assess..

[30] Ubeynarayana N, Paramsothy J, Bishop P, RobertoCalveloPereira R, Anderson CWN (2021). Effect of soil cadmium on root organic acid secretion by forage crops. Environ. Pollut..

[31] Barajas-Aceves M (2005). Comparison of different microbial biomass and activity measurement methods in metal-contaminated soils. Biores. Techn..

[32] Tsonev T, Cebola Lidon FJC (2012). Zinc in plants—An overview. Emir. J. Food Agric..

[33] Journal of Laws of the Republic of Poland item 1395. *Regulation of the Minister of the Environment (September 1, 2016) on the Method of Assessing the Pollution of the Earth’s Surface* (2016).

[34] Duan Y, Zhang Y, Zhao B (2022). Lead, zinc tolerance mechanism and phytoremediation potential of *Alcea rosea* (Linn.) Cavan and *Hydrangea macrophylla* (Thunb.) Ser. and ethylenediaminetetraacetic acid effect. Environ. Sci. Pollut. Res. Int..

[35] Longnecker, N. E. & Robson, A. D. Distribution and transport of zinc in plants. in *Zinc in Soils and Plants. Developments in Plant and Soil Sciences* (ed. Robson, A. D.). Vol. 55. (Springer, 1993).

[36] Du J, Zeng J, Ming X, He Q, Tao Q (2020). The presence of zinc reduced cadmium uptake and translocation in Cosmos bipinnatus seedlings under cadmium/zinc combined stress. Plant Physiol. Biochem..

[37] Dobrikova A, Apostolova E, Adamakis IDS, Hanć A, Sperdouli I (2022). Combined impact of excess zinc and cadmium on elemental uptake, leaf anatomy and pigments, antioxidant capacity, and function of photosynthetic apparatus in Clary Sage (*Salvia sclarea* L.). Plants.

[38] Barrutia O, Epelde JI, García-Plazaola C, Garbisu J, Becerri M (2009). Phytoextraction potential of two *Rumex acetosa* L. accessions collected from metalliferous and non-metalliferous sites: Effect of fertilization. Chemosphere.

[39] Yingping H, Xi Y, Gan L, Johnson D, Wu Y (2019). Effects of lead and cadmium on photosynthesis in *Amaranthus spinosus* and assessment of phytoremediation potential. Int. J. Phytorem..

[40] Dridi N, Bouslimi H, Caçador I, Sleimi N (2022). Lead tolerance, accumulation and translocation in two Asteraceae plants: *Limbarda crithmoides* and *Helianthus annuus*. S. Afr. J. Bot..

[41] Cherif J, Mediouni C, Ammar WB, Jemal F (2011). Interactions of zinc and cadmium toxicity in their efects on growth and in antioxidative systems in tomato plants *(Solanum lycopersicum*). J. Environ. Sci..

[42] Bayçu G, Gevrek-Kürüm N, Moustaka J, Csatári I, Rognes SE (2017). Cadmium-zinc accumulation and photosystem II responses of *Noccaea caerulescens* to Cd and Zn exposure. Environ. Sci. Pollut. Res..

[43] Dobrikova AG, Apostolova EL, Hanć A, Yotsova E, Borisova P (2021). Cadmium toxicity in *Salvia sclarea* L: An integrative response of element uptake, oxidative stress markers, leaf structure and photosynthesis. Ecotoxicol. Environ. Saf..

[44] Drinovec L, Drobne D, Jerman I, Zrimec A (2004). Delayed fluorescence of *Lemna minor*: A biomarker of the effects of copper, cadmium, and zinc. Bull. Environ. Contam. Toxicol..

[45] Morales M, Munné-Bosch S (2019). Malondialdehyde: Facts and artifacts. Plant Physiol..

[46] Caverzan A, Passaia G, Rosa B, Werner Ribeiro C (2012). Plant responses to stresses: Role of ascorbate peroxidase in the antioxidant protection. Genet. Mol. Biol..

[47] Mohamed AA, Castagna A, Ranieri A, di Toppi LS (2012). Cadmium tolerance in *Brassica juncea* roots and shoots is affected by antioxidant status and phytochelatin biosynthesis. Plant Physiol. Biochem..

[48] Cui E, Cui B, Fan X, Li S, Feng G (2021). Ryegrass (*Lolium multiflorum* L.) and Indian mustard (*Brassica juncea* L.) intercropping can improve the phytoremediation of antibiotics and antibiotic resistance genes but not heavy metals. Sci. Total Environ..

[49] Hultén, E. & Fries, M. *Atlas of North European Vascular Plants. North of the Tropic of Cancern. Introduction to Taxonomic index to the maps 1-996. Maps 1-996I*. (Koeltz Scientific Books, 1986).

[50] POWO. https://www.powo.science.kew.org/taxon/urn:lsid:ipni.org:names:18423-1 (2019).

[51] Ali H, Khan E, Sajad MA (2013). Phytoremediation of heavy metals—Concepts and applications. Chemosphere.

[52] Rezvani M, Faezeh Z (2011). Bioaccumulation and translocation factors of cadmium and lead in *Aeluropus littoralis*. Aust. J. Agric. Eng..

[53] Arnon D, Copper I (1949). Enzymes in isolated chloroplasts. Polyphenoloxidase in *Beta vulgaris*. Plant Physiol..

[54] Almeselmani M, Saud AAR, Hareri F, Al-nasan M, Ammar MA (2012). Physiological traits associated with drought tolerance of syrian durum wheat varieties under rainfed condition. Indian J. Plant Physiol..

[55] Creus CM, Sueldo RJ, Barass C (2004). Water relations and yield in *Azospirillum* inoculated wheat exposed to drought in the field. Can. J. Bot..

[56] Ostrowska-Gumkowska B, Ostrowska-Czubenko J (1991). Effect of comonomer content on thermal degradation of anionically modified poly(ethylene terephthalate). Eur. Polymer J..

[57] Patterson BD, Macrae EA, Ferguson IB (1984). Estimation of hydrogen peroxide in plant extracts using titanium(IV). Anal. Biochem..

[58] Aebi, H. & Catalase, E. *Methods of Enzymatic Analysis *(ed. Bergmeyer, H. U.)*. *273–286 (Chemie, 1983).

[59] Nakano Y, Asada K (1981). Hydrogen peroxide is scavenged by ascorbate-specific peroxidase in spinach chloroplasts. Plant Cell Physiol..

[60] Heath RL, Packer L (1968). Photoperoxidation in isolated chloroplasts: Kinetics and stoichiometry of fatty acid peroxidation. Arch. Biochem. Biophys..

[61] Bradford MM (1976). A rapid and sensitive method for the quantitation of microgram quantities of protein utilizing the principle of protein-dye binding. Anal. Biochem..

[62] Afzal M, Matsugo S, Sasai M, Xu B, Aoyama K, Takeuchi T (2003). Method to overcome photoreaction, a serious drawback to the use of dichlorofluorescin in evaluation of reactive oxygen species. Biochem. Biophys. Res. Commun..

