# Supplementary material for: Common weeds as heavy metal bioindicators: a new approach in biomonitoring
Source: Sci Rep. 2023 Apr 28;13:6926. doi: 10.1038/s41598-023-34019-9 (PMC10147616; doi:10.1038/s41598-023-34019-9)

Supplement materials

Common weeds as heavy metal bioindicators – a new approach in biomonitoring

Cakaj A.[1], Lisiak-Zielińska M.[1], Hanć A.[2], Małecka A.[3], Borowiak K.[1], Drapikowska M.[1*]

[1]*Department of Ecology and Environmental Protection, Faculty of Environmental and Mechanical Engineering, Poznań University of Life Sciences, Piątkowska 94C, 60-649, Poznań, Poland*

[2]*Department of Trace Analysis, Faculty of Chemistry, Adam Mickiewicz University, Uniwersytetu Poznańskiego 8, 61-614 Poznań, Poland*

[3]*Greater Poland Cancer Centre, The Cancer Epidemiology and Prevention Unit, Garbary 15 Street, 61-866 Poznan, Poland*

Email: [1]arlinda.cakaj@up.poznan.pl, [2]anettak@amu.edu.pl, [1]marta.lisiak@up.poznan.pl, [1]klaudia.borowiak@up.poznan.pl, [3]arleta.malecka@wco.pl , [1]maria.drapikowska@up.poznan.pl

Table S1. Heavy metals content (mg kg^{-1} DW) in analyzed species including research sites and plant organs (mean±SD)

**Plants**		**Cu**			**Zn**			**Cd**			**Pb**		
		**Soil**	**Leaves**	**Roots**	**Soil**	**Leaves**	**Roots**	**Soil**	**Leaves**	**Roots**	**Soil**	**Leaves**	**Roots**
Lolium multiflorum	1A	11.93±0.51	6.14±0.12^{d}	5.71±0.12^{ef}	26.31±0.63	33.92±1.44^{ghij}	111.58±0.67^{c}	0.16±0.01^{b}	0.12±0.01^{gh}	0.27±0.01^{fgh}	6.71±0.17	0.70±0.03^{bc}	1.21±0.02^{a}
	1B	17.97±0.32	8.30±0.12^{c}	6.33±0.19^{de}	29.16±1.16	29.27±3.50^{jk}	172.45±0.80^{a}	0.14±0.01^{b}	0.07±0.01^{hi}	0.26±0.01^{fghi}	7.49±0.08	0.59±0.02^{de}	1.19±0.01^{a}
	1C	11.29±0.51	7.77±0.51^{c}	7.23±0.09^{bcd}	24.51±1.31	81.13±9.85^{a}	161.85±0.82^{a}	0.13±0.03^{b}	0.18±0.06^{efg}	0.79±0.01^{c}	6.81±0.36	1.32±0.02^{a}	1.21±0.01^{a}
	Control	5.89±0.31	2.97±0.09^{jk}	2.56±0.11^{j}	15.78±0.34	20.71±2.15^{l}	57.41±0.32^{e}	0.09±0.01^{b}	0.04±0.01^{i}	0.18±0.01^{hijkl}	4.89±0.13	0.41±0.02^{gh}	0.56±0.01^{de}
Trifolium pratense	2A	11.91±0.54	4.56±0.17^{fghi}	6.34±0.08^{de}	26.38±0.50	34.37±0.38^{fghij}	31.07±0.54^{ghi}	0.15±0.01^{b}	0.08±0.01^{hi}	0.32±0.01^{ef}	6.70±0.17	0.31±0.03^{ij}	0.72±0.02^{c}
	2B	17.87±0.43	5.86±0.24^{ed}	9.20±0.14^{a}	29.19±1.01	38.00±2.13^{efg}	80.57±1.15^{d}	0.15±0.02^{b}	0.06±0.01^{hi}	0.25±0.01^{fghi}	7.51±0.06	0.63±0.01^{cd}	0.53±0.03^{de}
	2C	11.25±0.54	20.38±0.14^{a}	8.00±0.41^{bc}	24.48±1.38	68.49±0.26^{b}	25.65±1.53^{hi}	0.11±0.02^{b}	0.44±0.01^{c}	0.22±0.01^{ghij}	6.92±0.41	0.36±0.01^{hi}	0.57±0.02^{d}
	Control	5.21±0.21	3.82±0.11^{hij}	3.85±0.05^{hi}	15.03±0.26	24.61±0.25^{kl}	21.31±0.71^{hi}	0.09±0.01^{b}	0.05±0.01^{hi}	0.15±0.02^{ijkl}	4.92±0.16	0.23±0.03^{jk}	0.37±0.01^{fg}
Rumex accetosa	3A	11.98±0.49	4.99±0.13^{fg}	7.99±0.02^{bc}	26.29±0.62	29.35±0.37^{jk}	22.87±0.27^{hi}	0.16±0.01^{b}	0.11±0.01^{hi}	0.08±0.01^{l}	6.74±0.15	0.59±0.01^{de}	0.46±0.01^{ef}
	3B	17.99±0.32	4.37±0.11^{ghi}	9.18±0.02^{a}	29.11±1.19	37.77±0.43^{efg}	32.92±0.54^{ghi}	0.13±0.02^{b}	0.21±0.01^{e}	0.15±0.01^{jkl}	7.45±0.13	0.18±0.01^{k}	0.21±0.01^{ij}
	3C	11.25±0.55	10.51±0.16^{b}	9.66±0.13^{a}	24.50±1.30	49.79±0.36^{d}	44.95±0.19^{efg}	0.14±0.01^{b}	0.20±0.01^{ef}	0.21±0.01^{ghij}	6.79±0.38	0.75±0.05^{b}	0.99±0.02^{b}
	Control	5.43±0.20	4.01±0.21^{hi}	5.32±0.09^{efg}	16.13±0.38	18.56±0.41^{l}	17.65±0.19^{i}	0.09±0.02^{b}	0.12±0.01^{ghi}	0.10±0.01^{kl}	5.09±0.13	0.21±0.03^{k}	0.22±0.04^{hij}
Alcea rosea	4A	11.91±0.49	6.11±0.06^{d}	6.15±0.34^{e}	26.33±0.66	39.52±0.81ef	135.85±2.25^{b}	0.17±0.02^{b}	0.58±0.04^{b}	0.79±0.05^{c}	6.71±0.16	0.58±0.01^{de}	0.31±0.01^{ghi}
	4B	17.97±0.36	4.74±0.23^{fgh}	8.13±0.06^{b}	29.16±1.16	55.73±3.03^{c}	131.93±0.99^{b}	0.14±0.01^{b}	0.58±0.01^{b}	1.24±0.01^{a}	7.47±0.09	0.34±0.01^{hi}	0.32±0.01^{gh}
	4C	11.25±0.54	5.22±0.09^{ef}	5.71±0.20^{ef}	24.57±1.28	36.89±1.22^{efgh}	53.77±1.22^{ef}	0.13±0.01^{b}	0.34±0.01^{d}	0.19±0.01^{hijk}	6.82±0.35	0.49±0.02^{ef}	0.73±0.02^{c}
	Control	4.16±0.49	2.57±0.94^{k}	3.31±0.95^{ij}	16.08±0.91	31.33±1.83^{hij}	58.16±1.62^{e}	0.11±0.03^{b}	0.20±0.02^{efg}	0.31±0.08^{efg}	5.17±0.11	0.24±0.05^{jk}	0.22±0.14^{hij}
Amaranthus retroflexus	5A	11.89±0.55	4.65±0.07^{fghi}	3.10±0.16^{ij}	26.14±0.71	52.62±0.76^{cd}	23.62±1.27^{hi}	0.18±0.03^{b}	0.19±0.01^{efg}	0.21±0.01^{ghijk}	6.69±0.12	0.53±0.0^{1ef}	0.45±0.01^{ef}
	5B	17.91±0.38	4.10±0.02^{hi}	4.37±0.10^{gh}	29.16±1.13	39.63±0.54^{e}	37.77±0.65^{fgh}	0.13±0.01^{b}	0.12±0.01^{gh}	0.21±0.01^{ghij}	7.53±0.10	0.55±0.01^{def}	0.18±0.01^{i}
	5C	11.21±0.59	4.38±0.04^{ef}	3.74±0.15^{ef}	27.73±0.91	46.12±0.61^{efghi}	30.69±0.91^{ef}	0.14±0.03^{b}	0.33±0.01^{d}	0.21±0.01^{hijk}	7.07±0.09	0.47±0.01^{fg}	0.73±0.01^{c}
	Control	4.19±0.32	2.23±0.03^{k}	2.19±0.07^{j}	15.87±0.42	30.14±0.95^{ijk}	17.40±0.86^{i}	0.13±0.06^{b}	0.12±0.03^{ghi}	0.21±0.03^{ghji}	4.31±0.22	0.18±0.03^{k}	0.16±0.02^{i}
Plantago lanceolata	6A	11.81±0.60	3.96±0.05^{hi}	7.21±0.18^{cd}	26.28±0.66	36.71±1.36^{efgh}	99.21±1.66^{c}	0.16±0.02^{b}	0.24±0.02^{e}	1.11±0.01^{b}	6.73±0.15	0.39±0.01^{hi}	0.29±0.02^{ghij}
	6B	17.97±0.34	4.42±0.11^{fghi}	5.51±0.04^{ef}	29.11±1.21	31.75±1.56^{hij}	77.40±0.13^{d}	0.15±0.01^{b}	0.43±0.01^{c}	0.28±0.01^{fgh}	7.49±0.08	0.48±0.01^{fg}	0.77±0.01^{c}
	6C	11.25±0.53	7.67±0.29^{c}	4.94±0.06^{fg}	24.53±1.29	80.45±7.70^{a}	114.77±0.94^{c}	0.12±0.01^{b}	0.51±0.02^{b}	0.41±0.01^{e}	6.77±0.37	0.62±0.01^{d}	0.27±0.02^{ghij}
	Control	4.15±0.37	3.78±0.41^{ij}	3.14±0.64^{ij}	16.32±0.53	20.60±1.83^{l}	38.56±3.31^{efgh}	0.26±0.02^{a}	0.69±0.04^{a}	0.64±0.04^{d}	4.35±0.17	0.35±0.03^{hi}	0.25±0.03^{ghij}

where identical superscripts (a, b, c) denote non-significant differences between means in columns according to the post-hoc Scheffé test

Table S2. Two-way ANOVA for all analysed HMs levels in all materials
(F statistic for interaction *city* x *research site*)

	**Cu**	**Zn**	**Cd**	**Pb**
**soil**	1.81	0.15	10.79*	1.60
**leaves**	962.52*	528.6*	285.55*	400.85*
**roots**	89.92*	231.02*	706.49*	338.88*

where * means significant influence ($\alpha \leq 0.05$)

Table S3. Air pollution at research site A and B during exposition (mean±SD)

Date	Research site A			Research site B		
	PM_{10} [ug/m^3]	$PM_{2.5}$ [ug/m^3]	NO_2 [ug/m^3]	PM_{10} [ug/m^3]	$PM_{2.5}$ [ug/m^3]	NO_2 [ug/m^3]
2021-05-31	16.626±5.476	7.022±2.462	16.804±20.634	8.891±2.946	7.022±2.462	25.678±29.264
2021-06-01	28.908±8.944	12.613±2.956	25.963±14.829	19.642±2.947	14.529±2.638	27.033±11.464
2021-06-02	27.375±9.641	12.667±4.071	13.058±5.82	20.708±3.267	16.063±3.279	18.196±8.589
2021-06-03	19.446±4.854	10.391±3.101	9.004±6.383	18.7±2.824	15.121±2.564	14.813±11.952
2021-06-04	24.888±6.695	11.667±3.837	17.642±10.242	23.933±5.105	16.125±2.998	27.550±21.340
2021-06-05	22.213±4.782	12.983±4.187	15.625±13.968	22.313±4.782	16.733±3.895	27.175±24.725
2021-06-06	20.967±6.489	12.867±4.253	11.904±10.454	20.788±6.073	15.700±3.988	24.933±28.073
2021-06-07	31.763±12.157	15.417±3.943	18.413±16.18	23.296±4.41	17.829±3.918	27.329±23.353
2021-06-08	25.975±7.820	10.467±3.084	13.246±10.958	16.208±3.77	12.071±3.053	16.558±15.265
2021-06-09	26.954±21.274	6.758±2.047	9.629±6.418	13.588±2.671	9.696±1.626	13.363±16.631
2021-06-10	30.325±23.544	11.583±5.418	27.896±17.824	19.517±4.541	13.875±5.175	34.613±21.453
2021-06-11	20.988±10.791	10.539±2.593	24.040±18.873	17.696±2.521	12.700±2.267	30.088±25.911
2021-06-12	26.642±17.553	13.417±7.040	21.063±18.272	17.421±7.617	13.500±7.118	23.542±16.479
2021-06-13	12.242±1.364	5.654±1.057	2.929±1.858	10.183±1.548	6.154±1.078	7.063±3.105
2021-06-14	19.038±4.632	10.254±1.710	12.974±7.257	18.625±2.626	13.946±1.502	18.438±10.354
2021-06-15	35.196±22.179	13.674±2.796	15.691±6.438	19.158±2.383	13.717±2.754	17.271±10.004
2021-06-16	16.065±8.187	6.014±1.543	8.668±5.087	13.579±4.553	7.421±3.079	12.158±5.772
2021-06-17	36.971±19.675	16.433±5.184	11.979±4.615	26.079±6.130	15.771±3.068	17.163±8.077
2021-06-18	35.083±14.435	15.517±2.280	11.900±6.652	27.750±7.000	16.029±6.147	21.888±18.438
2021-06-19	30.779±7.414	19.491±5.890	10.670±5.724	30.208±10.198	20.363±9.481	18.321±12.064
2021-06-20	27.208±8.661	12.942±3.181	5.142±2.319	21.996±4.317	12.792±3.923	10.979±4.257
2021-06-21	36.530±17.782	14.352±4.081	10.835±5.103	27.726±8.341	14.339±4.325	17.617±9.700
2021-06-22	33.046±16.840	19.25±8.566	15.354±7.257	31.446±9.440	18.779±4.250	16.329±6.747
2021-06-23	19.246±19.774	8.548±3.002	8.426±5.035	19.750±3.950	10.346±2.039	11.863±7.815
2021-06-24	27.229±18.552	13.717±5.248	14.996±7.196	25.504±9.492	12.158±3.010	16.467±7.088
2021-06-25	17.513±10.156	8.513±2.493	11.917±9.664	16.667±5.314	10.558±3.485	15.271±10.226
2021-06-26	18.154±5.799	12.467±5.244	12.950±8.904	23.246±9.265	12.996±5.256	15.008±7.463
2021-06-27	10.792±4.290	6.775±2.901	10.725±11.376	12.050±4.608	8.433±3.471	19.763±21.002
2021-06-28	22.792±9.508	9.361±2.780	22.991±14.779	16.879±5.817	9.983±4.404	26.983±18.988
2021-06-29	29.154±12.027	12.525±3.403	22.579±14.223	25.088±8.450	13.975±5.572	30.954±19.609
2021-06-30	16.742±6.998	7.904±3.445	12.308±3.983	16.725±7.188	11.388±4.447	16.883±6.681
2021-07-01	19.729±14.018	7.163±4.002	8.146±2.764	13.700±7.373	8.129±4.321	11.729±3.040
2021-07-02	26.975±15.869	10.261±3.670	12.543±4.641	18.192±4.786	11.142±3.269	15.988±5.756
2021-07-03	16.563±7.391	7.279±2.257	7.354±5.305	16.167±4.727	10.258±3.843	14.629±13.483
2021-07-04	13.508±1.681	8.675±1.187	8.908±8.127	17.325±3.954	11.871±3.200	20.071±22.582
2021-07-05	39.292±52.727	14.813±5.627	20.813±10.568	24.125±5.072	16.346±3.514	26.246±14.852
2021-07-06	27.408±11.942	8.539±3.417	15.657±12.353	15.521±5.808	9.208±6.403	23.729±17.488
2021-07-07	44.058±53.934	8.65±5.646	13.338±7.348	11.438±4.345	7.104±3.004	16.483±15.665
2021-07-08	16.533±5.492	9.788±2.977	8.133±2.209	18.625±5.975	9.367±2.070	9.583±1.851
2021-07-09	13.208±4.037	8.354±2.316	10.146±4.723	16.254±3.651	9.613±1.723	14.304±6.412
2021-07-10	11.392±3.633	5.957±2.355	8.717±11.057	12.258±4.469	8.021±2.989	11.746±13.055
2021-07-11	19.267±2.636	12.133±2.817	7.238±4.438	24.683±4.113	16.250±5.067	14.033±10.987

Figure S1. Hydrogen peroxide level (nMol $H_2O_2 \times min^{-1} \times$ mg $protein^{-1}$) in leaves and roots of tested species samples, abbreviations are given in Table 1.

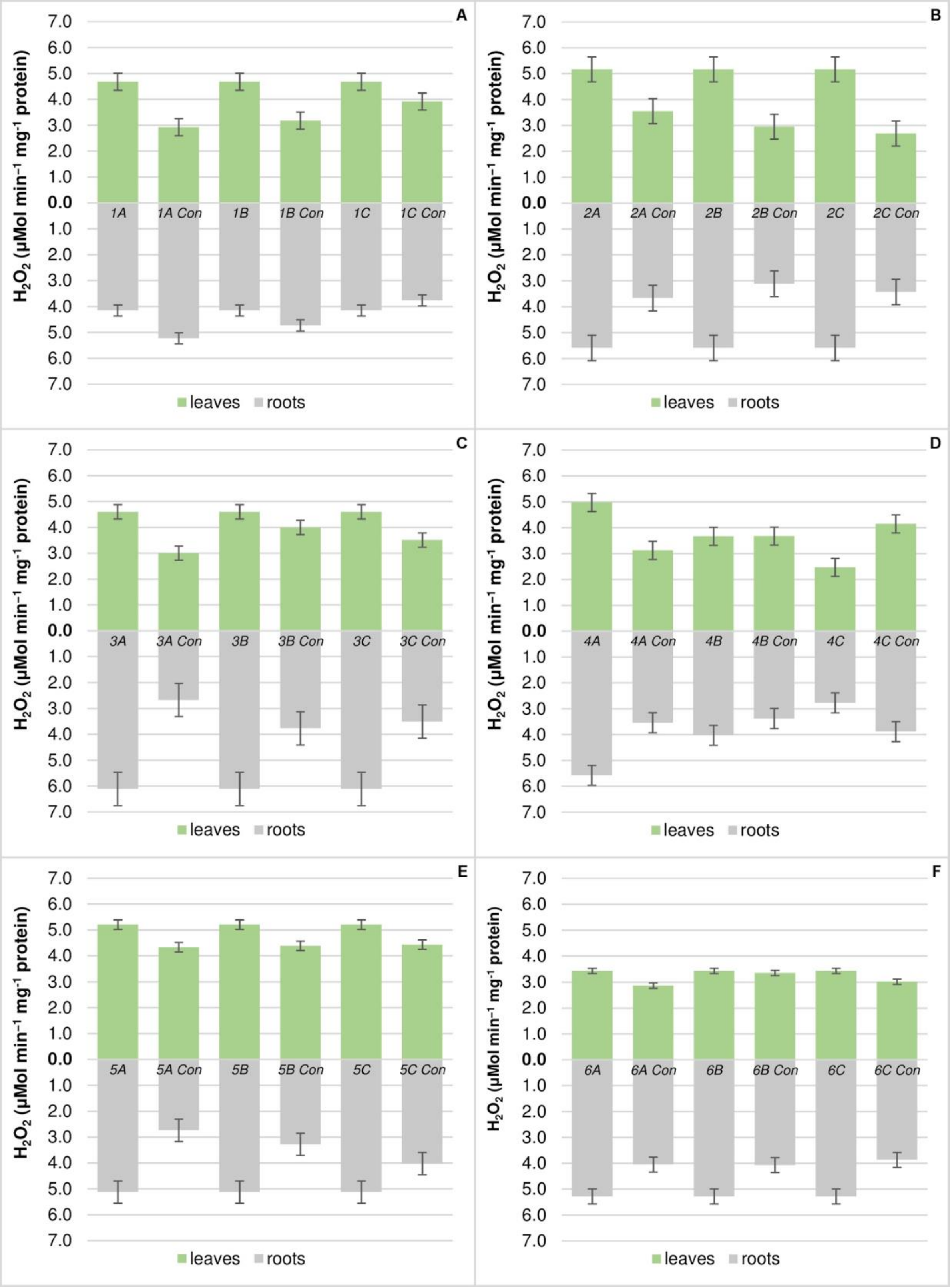

Figure S2. CAT (μMol min^{-1} mg^{-1} protein) activities in leaves and roots of examined species samples, abbreviations are given in Table 1.

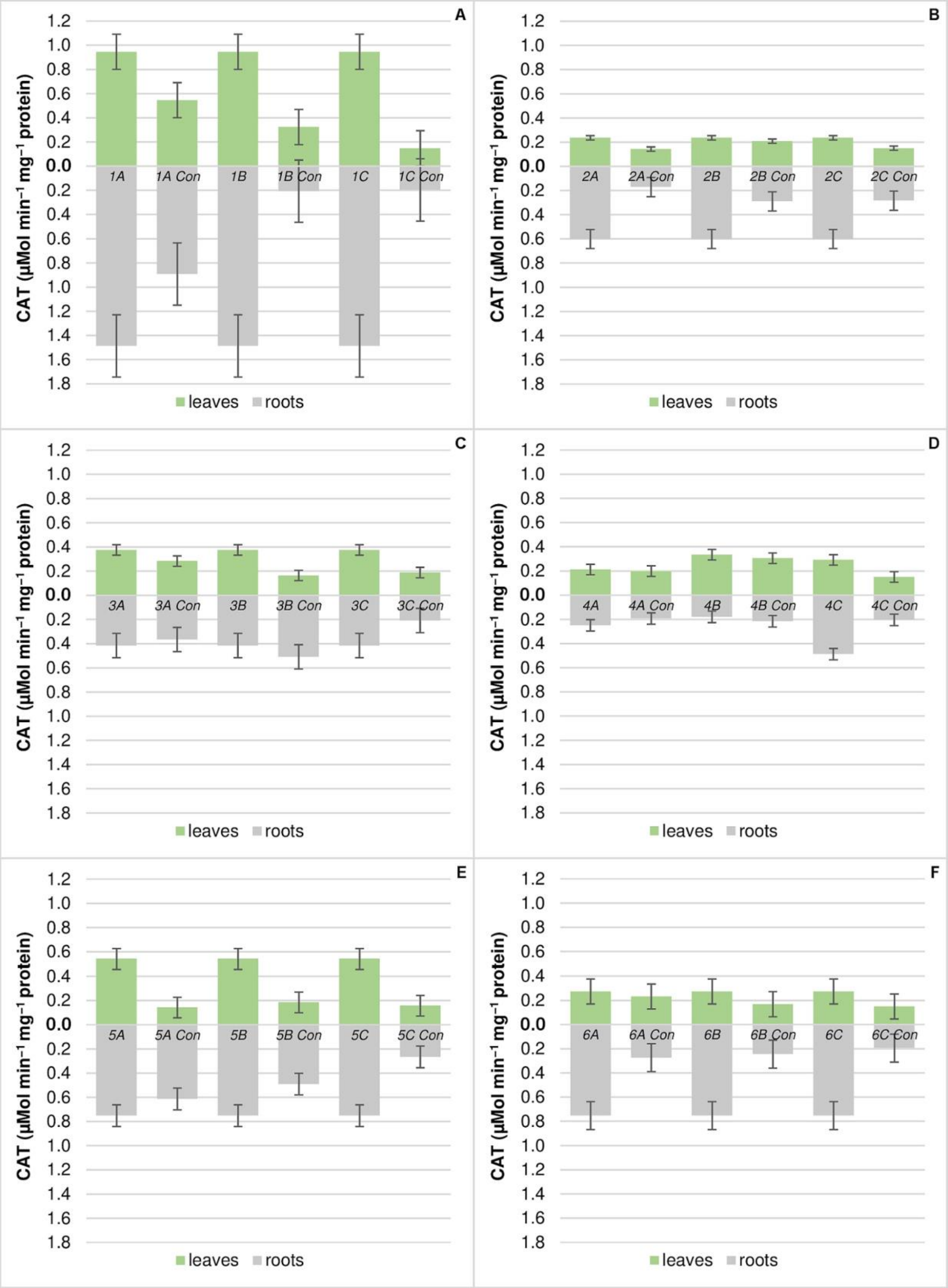

Figure S3. APOX (μMol min^{-1} mg^{-1} protein) activities in leaves and roots of examined species samples, abbreviations are given in Table 1.

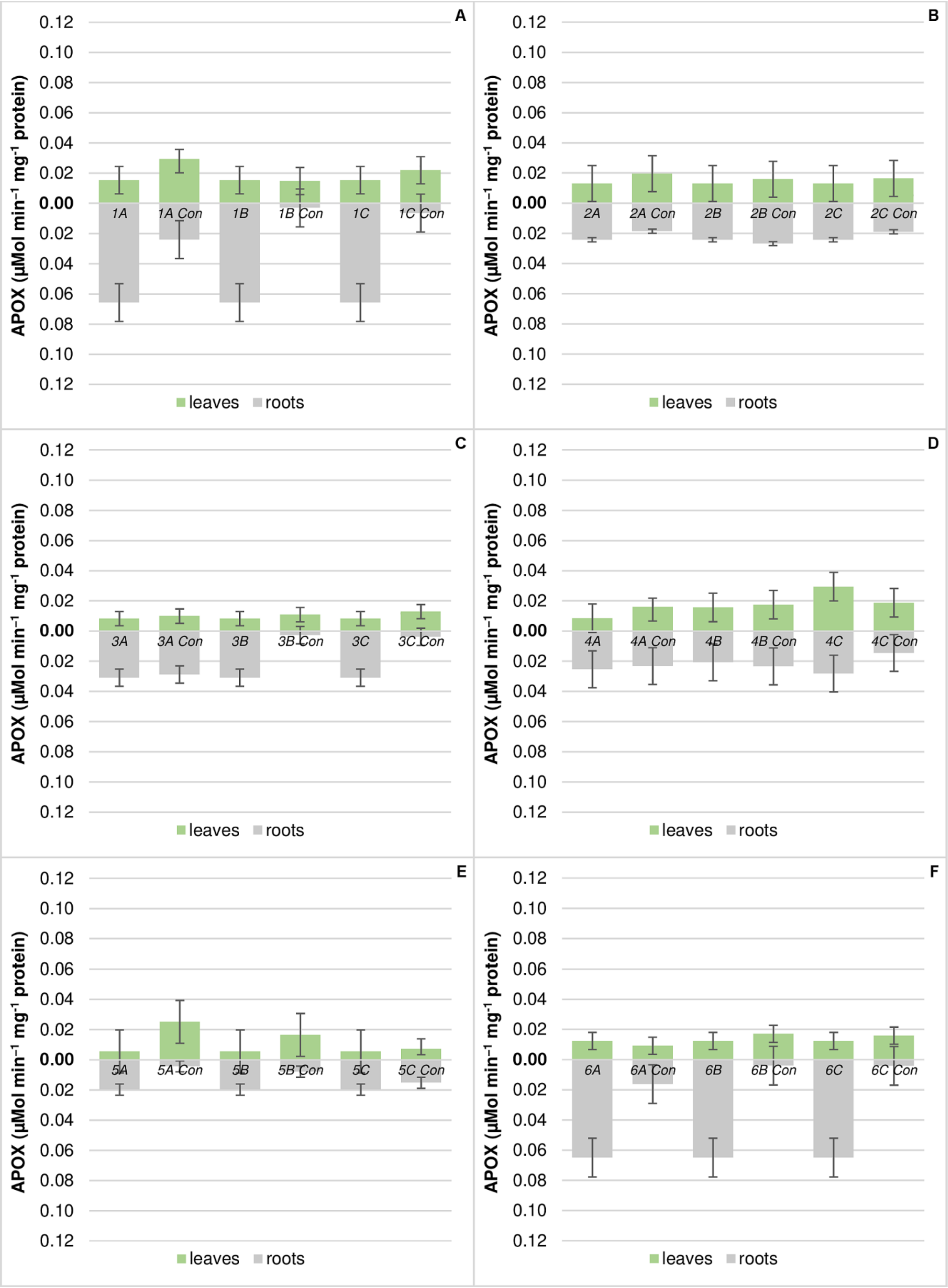

Figure S4. MDA (μMol min^{-1} mg^{-1} protein) activities in leaves and roots of examined species samples, abbreviations are given in Table 1.

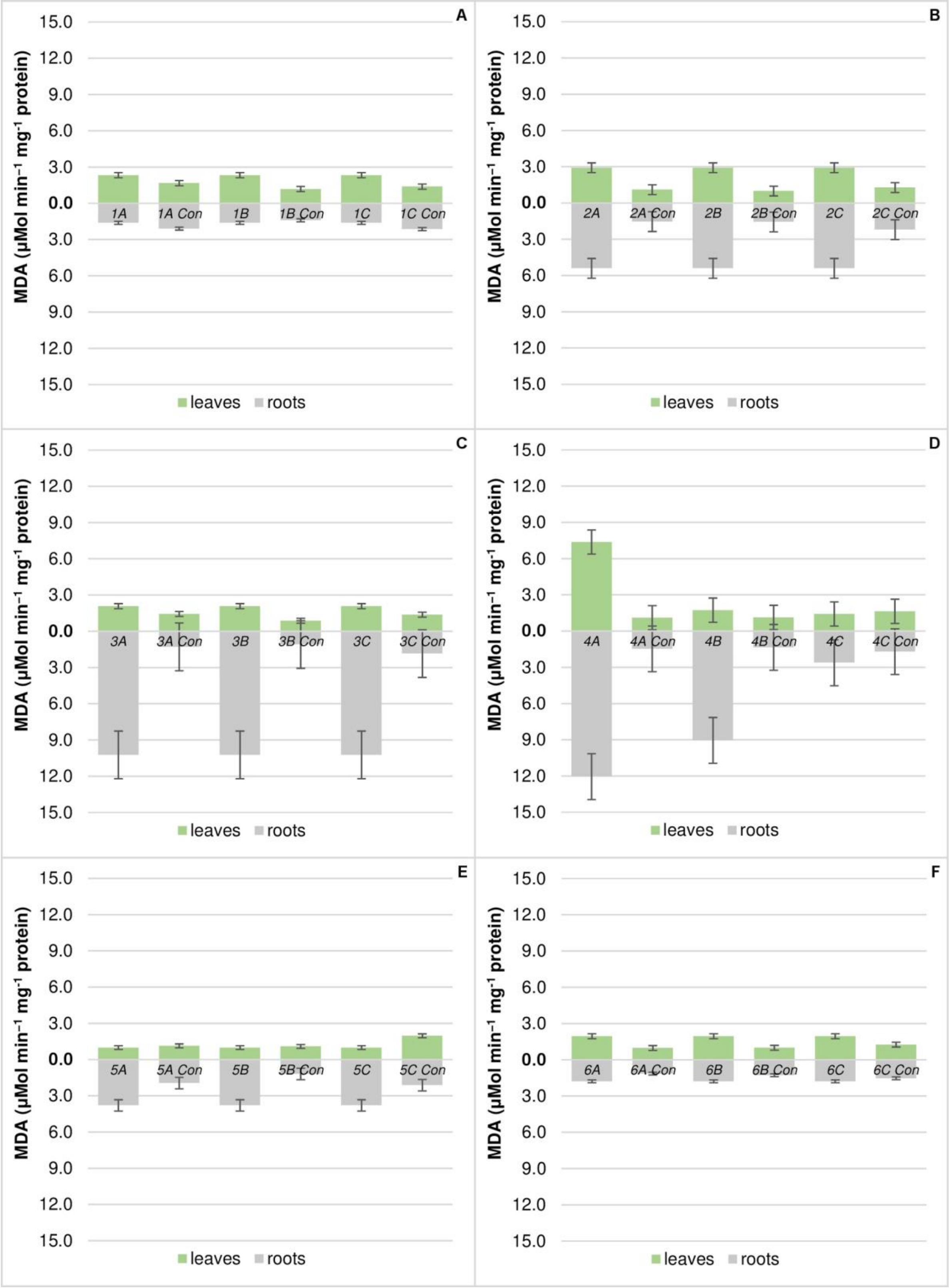

Supplement: Supplementary file 1 — Supplementary Information. [file 41598_2023_34019_MOESM1_ESM.pdf]
